# Projected amplification of food web bioaccumulation of MeHg and PCBs under climate change in the Northeastern Pacific

**DOI:** 10.1038/s41598-018-31824-5

**Published:** 2018-09-07

**Authors:** Juan José Alava, Andrés M. Cisneros-Montemayor, U. Rashid Sumaila, William W. L. Cheung

**Affiliations:** 1 Institute for the Oceans and Fisheries, University of British Columbia, AERL 2202 Main Mall, Vancouver, BC V6T 1Z4 Canada; 2 School of Public Policy and Global Affairs, University of British Columbia, 6476 NW Marine Drive, Vancouver, BC V6T 1Z2 Canada

## Abstract

Climate change increases exposure and bioaccumulation of pollutants in marine organisms, posing substantial ecophysiological and ecotoxicological risks. Here, we applied a trophodynamic ecosystem model to examine the bioaccumulation of organic mercury (MeHg) and polychlorinated biphenyls (PCBs) in a Northeastern Pacific marine food web under climate change. We found largely heterogeneous sensitivity in climate-pollution impacts between chemicals and trophic groups. Concentration of MeHg and PCBs in top predators, including resident killer whales, is projected to be amplified by 8 and 3%, respectively, by 2100 under a high carbon emission scenario (Representative Concentration Pathway 8.5) relative to a no-climate change control scenario. However, the level of amplification increases with higher carbon emission scenario for MeHg, but decreases for PCBs. Such idiosyncratic responses are shaped by the differences in bioaccumulation pathways between MeHg and PCBs, and the modifications of food web dynamics between different levels of climate change. Climate-induced pollutant amplification in mid-trophic level predators (Chinook salmon) are projected to be higher (~10%) than killer whales. Overall, the predicted trophic magnification factor is ten-fold higher in MeHg than in PCBs under high CO_2_ emissions. This contribution highlights the importance of understanding the interactions with anthropogenic organic pollutants in assessing climate risks on marine ecosystems.

## Introduction

While persistent organic pollutants (POPs) and mercury contamination of the marine environment have long been recognized as global problems^1–3^, the effects of climate change in exacerbating pollutant toxicity and bioaccumulation have recently drawn attention from eco-toxicological research arenas^4–11^. MeHg and PCBs are amongst the most prevalent chemicals in marine food webs, affecting the health of marine organisms^12,13^. Contaminated seafood poses significant health risks for human consumption, particularly in communities relying heavily on marine foods as a source of nutrition and socio-economic wellbeing^1,14,15^.

Ocean warming, deoxygenation and ocean acidification amplify these impacts by increasing the exposure and bioaccumulation rates for contaminants in marine food webs^1,6,7,16^. These interactions include phenomena considered either climate change dominant (i.e., climate change leads to an increase in contaminant exposure/sensitivity) or contaminant dominant (i.e., contamination leads to an increase in climate change susceptibility)^1,11^. Such climate-pollution interactions are already evidenced in sensitive species and marine ecosystems from the Arctic and Antarctic regions^1,6,16,17^. For example, polar bears (*Ursus maritimus*) and sea birds from a warming Arctic exhibit increasing concentrations of some POPs^6^ and mercury in sea birds^17^ because of changes in their diet composition and food web structure, and alteration in pollutants exposure and pathways, driven by climate change^6,17^. Similarly, concentrations of dichlorodiphenyltrichloroethanes (DDTs) have not decreased in Adelie penguins (*Pygoscelis adeliae*) for more than three decades in the western Antarctic Peninsula, where melting glaciers are considered as a possible recurrent source of DDT contamination to the regional marine food web, upon which penguins rely on^16^. However, information on the extent to which non-Arctic marine ecosystems are affected is limited^1^.

To analyze the potential synergistic impacts of climate change on pollutant bioaccumulation, we simulated forward-looking scenarios for the bioaccumulation of MeHg and PCBs (i.e. as the sum of congeners, ∑PCB; please see Supplementary Information) in a regional marine food web in the Northeastern Pacific (Supplementary Figs S1, S2 and Table S1) by using the Ecotracer module in Ecopath with Ecosim (EwE) (see Methods; Supplementary Fig. S3). The model accounts for the flow of biomasses and contaminants across 20 species groups in the ecosystem through their trophic interactions (Fig. S2 and Table S1), and includes the mean temperature tolerance (MTT) in marine invertebrates and fish (Table S2) and contaminant input data (Tables S3 and S4), but it does not account for some pollutant biogeochemical processes such as PCB cycling and increased rates of mercury methylation in a warming environment. We hypothesize that bioaccumulation of organic pollutants, specifically MeHg and PCBs, can be amplified in the food web by climate change and that the level of bioamplification may vary between different pollutants.

Interactions between biogeochemical climate change drivers and pollutants were modelled through deterministic simulations under climate change forcing scenarios, including Representative Concentrations Pathway (i.e. RCP 2.6-‘strong mitigation’/low CO_2_ emissions and RCP 8.5-‘business-as-usual’/high CO_2_ emissions), and compared against a reference baseline (control) scenario with no-climate change forcing, i.e. no changes in CO_2_ levels and biogeochemical variables from the present conditions (Table 1). Our model represented the effects of the changes in sea surface temperature, dissolved oxygen level and pH (i.e. ocean acidification) on the metabolic efficiency of functional groups, resulting in increased or decreased biological productivity (see Supplementary Information). Metabolic efficiency was determined by the ratio of consumption to production of each functional group that changed dynamically as trophic interactions were modified by the climatic forcing. Projected changes in primary production were represented by changing phytoplankton biomass (i.e. phytoplankton forcing function under either RCP 2.6 or RCP 8.5) in the ecosystem model (see Supplementary Information). Changes in ocean variables were based on outputs from the NOAA’s Geophysical Fluid Dynamic Laboratory Earth System Model 2M (NOAA-GFDL ESM2M) (see Methods; Supplementary Information; Figs S4 and S5).

**Table 1:** Relative differences (%) reported as mean and 95% Confidence Intervals (CI) between climate change scenarios (i.e. RCP 2.6* and RCP 8.5**) relative to the baseline (i.e. no climate change control scenario) for the simulation period 2005–2099 in representative functional groups or/and species of the marine food web from the Northeastern Pacific.

Functional Group/Species	Scenarios	Mercury (CH_3_Hg)		PCBs	
		Mean	95% CI	Mean	95% CI
Phytoplankton	RCP2.6/Baseline	2.7	1.17	19	1.64
	RCP8.5/Baseline	12.0	1.16	11	1.01
Zooplankton	RCP2.6/Baseline	3.1	1.06	20	1.55
	RCP8.5/Baseline	12.4	1.07	12	0.90
Foragefish	RCP2.6/Baseline	3.3	0.96	20	1.53
	RCP8.5/Baseline	14	0.98	13	0.88
Squid	RCP2.6/Baseline	4.3	1.11	21	1.60
	RCP8.5/Baseline	15	1.21	15	1.06
Chinook salmon	RCP2.6/Baseline	1.2	0.77	17.5	1.40
	RCP8.5/Baseline	10	0.82	10	0.84
Southern resident killer whale	RCP2.6/Baseline	0.90	0.18	0.35	0.23
	RCP8.5/Baseline	8.20	0.38	2.60	0.17

*RCP 2.6: ‘strong mitigation’/‘low CO_2_ emissions’.

**RCP 8.5: ‘business-as-usual’/‘high CO_2_ emissions’.

We accounted for model uncertainties associated with the biases between observed and predicted contaminant levels as well as the internal variability of the projections (Supplementary Information; Tables S5–S7). Specifically, model bias (*MB*) and *MB* standard deviation (i.e. *MB*_*SD*_, an indicator of variability and uncertainty of trophodynamic model predictions) were calculated by using projections of historical concentrations of PCBs and MeHg (i.e. from 1930–2010) simulated in southern resident killer whales, *Orcinus orca* (SRKW hereafter), and Chinook salmon (*Oncorhynchus tshawytscha*) with Ecotracer, and empirical PCB data reported for these species (see Methods; Supplementary Information). This is done by comparing the simulated contaminant data to the empirical contaminant data observed in these species to test the performance of the modelling approach and reveal any underlying variations linked to contaminant concentrations and lipid content reported in the literature.

## Results

**Figure 1:**
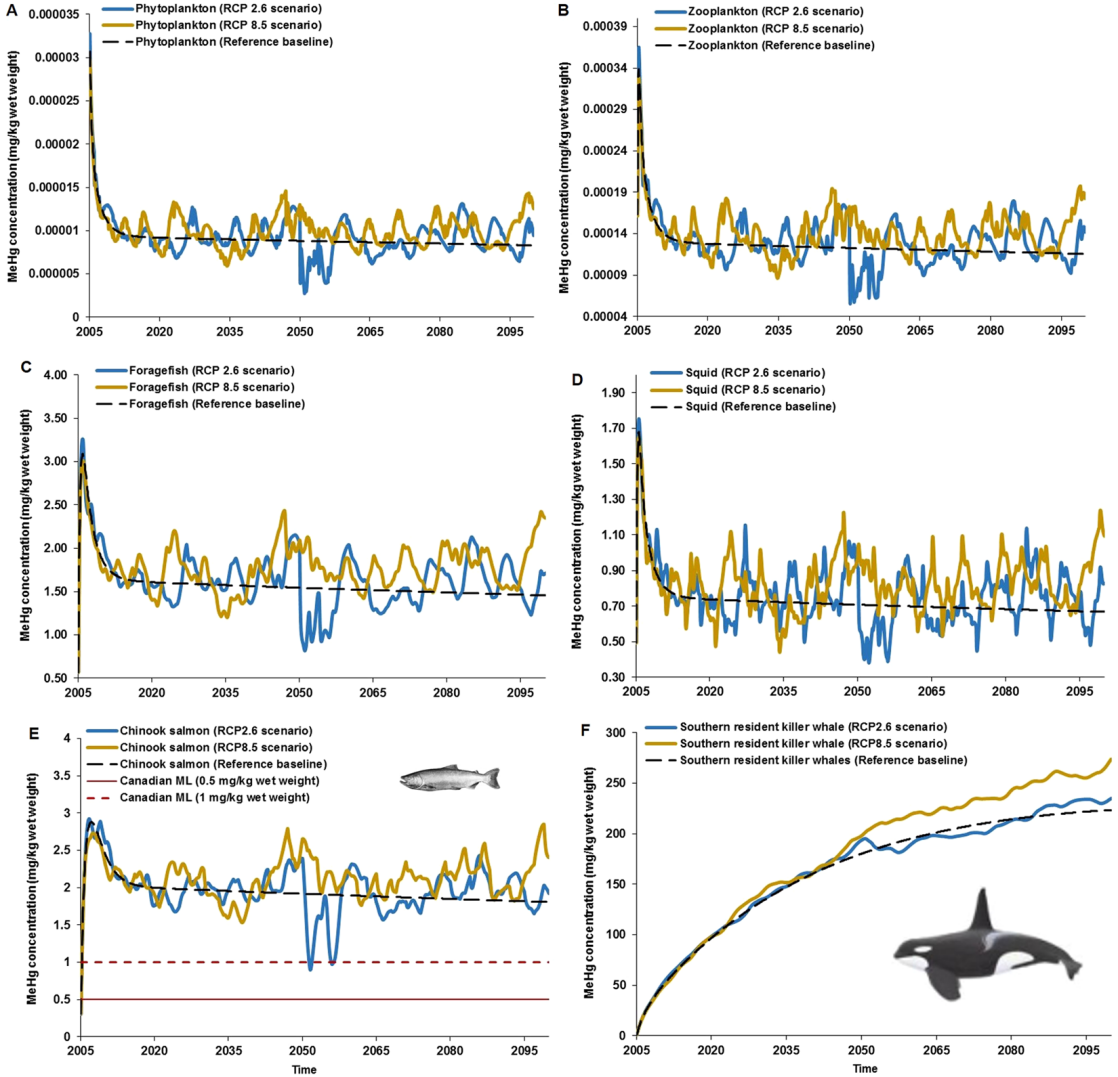
Projections of MeHg concentrations in the Chinook salmon-southern resident killer whale food web of the Northeastern Pacific. Simulations are shown for (**A**) phytoplankton, (**B**) zooplankton, (**C**) foragefish (i.e. functional group that includes Pacific sardine,* Sardinops sagax* *caeruleus*; California/northern anchovy, *Engraulis* *mordax*; Pacific herring, *Clupea pallasi*; sand lance, *Ammodytes hexapterus*; eulachon, *Thaleichthys pacificus*; American shad, *Alosa sapidissima*; surf smelt, *Hypomesus* *pretiosus*; whitebait smelt, *Allosmerus elongates*), (**D**) squid (*Loligo*), (**E**) Chinook salmon and (**F**) southern resident killer whales under RCP 2.6 and 8.5 scenarios and the reference baseline/no-climate change control scenario (black dashed line). For the MeHg predictions in Chinook salmon (panel E), the red solid and dashed lines represent the Canada’s maximum level (ML) mercury consumption limits of 0.5 mg/kg and 1.0 mg/kg wet weight, respectively. Public health implications concerning to the projected MeHg concentrations in salmon is available in the Supplementary Information.

**Figure 2:**
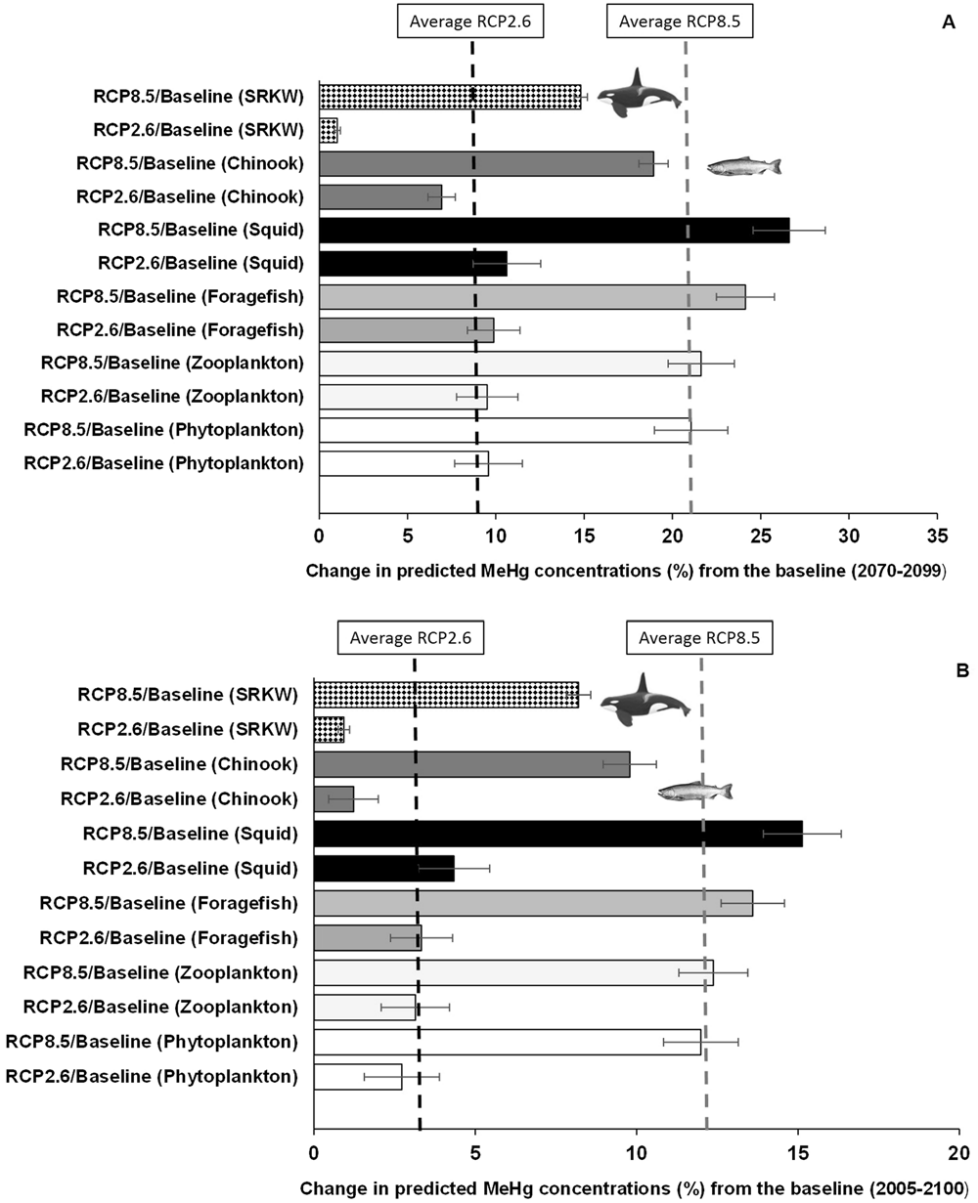
Mean percentage change in MeHg concentrations projected in the food web from 2070 to 2099 (**A**) and for the entire simulation period (2005–2099) (**B**). Bars represent the mean ± 95% CI of changes under RCP 2.6 and RCP 8.5 scenarios relative to the reference baseline (i.e. no-climate change control) for phytoplankton, zooplankton, foragefish, squid, Chinook salmon and southern resident killer whales (SRKW). The black and grey dashed lines represent the average MeHg concentrations from 2070–2099 and from 2005–2099 under the ‘business-as-usual’ (RCP 8.5) and ‘strong mitigation’ (RCP 2.6) scenarios, respectively.

Our model projected climate-amplification of MeHg concentrations in the food web, with varying sensitivities among functional groups (Figs 1A–F and 2). Average concentrations of MeHg were higher under both RCP 2.6 and RCP 8.5 by the 2080 s (average of 2070–2099, representing the end of the 21^st^ century) relative to the control scenario, with the level of amplification being higher under the ‘business-as-usual’ (8% and 20% under RCP 2.6 and RCP 8.5, respectively; Fig. 2A,B and Table 1).

Amongst the functional groups, MeHg bioaccumulation in Chinook salmon and SRKW (Fig. 1E,F) is more sensitive to climate change (i.e. species-specific response to the effect of climate change forcing) relative to other lower trophic level groups (Fig. 1A–D). While Chinook salmon showed high concentration pulses under RCP 8.5, SRKW exhibited an increasing, smooth trend in MeHg concentrations until reaching steady state toward the end of the 21^st^ century (Fig. 1F). Although MeHg concentrations tend to reach steady state in plankton over time, the projections for other low trophic level species unveiled increasing concentrations at the very beginning of the simulation, followed by a rapid decline with temporal pulses in the long term (Fig. 1A–E).

**Figure 3:**
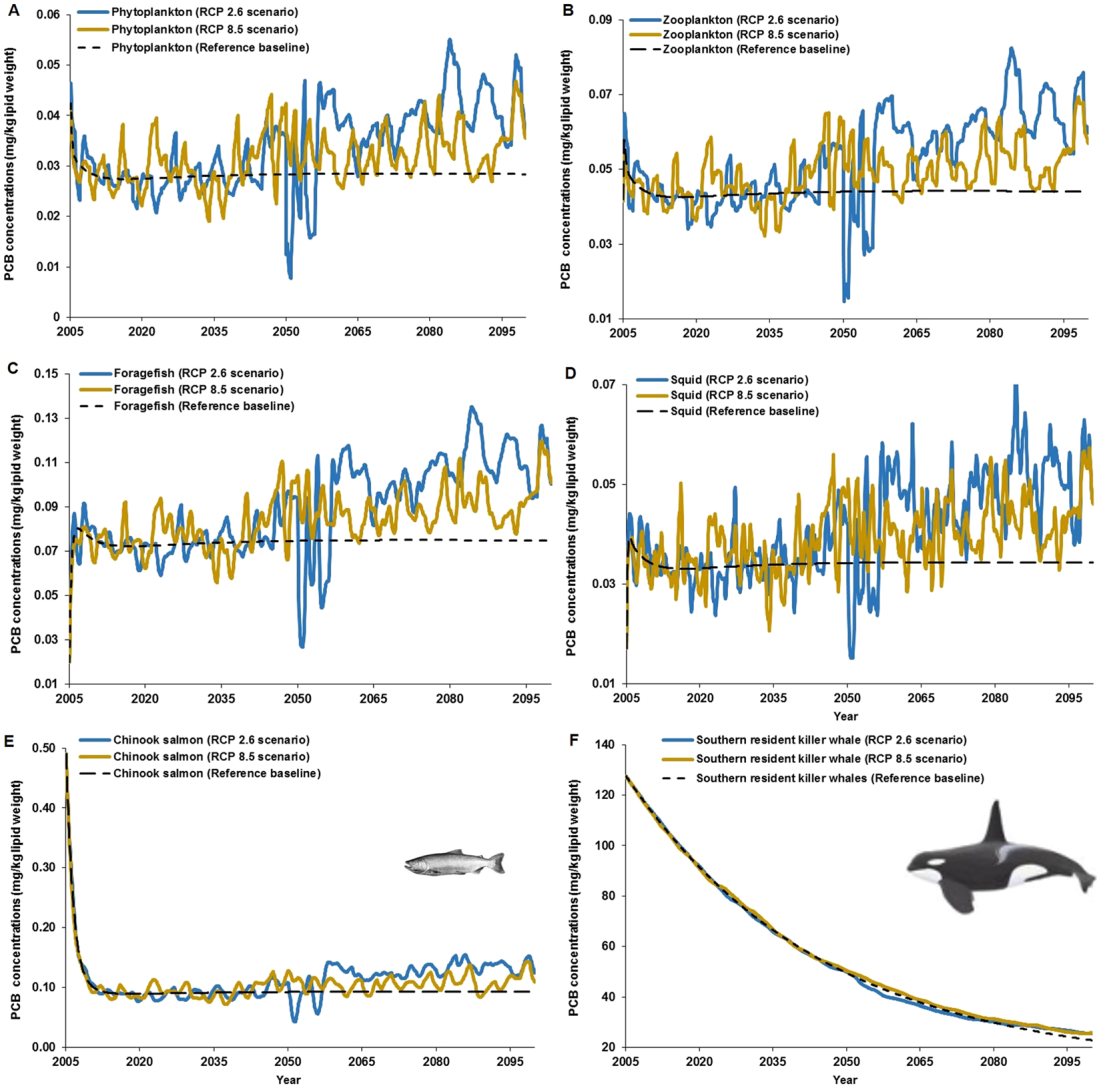
Projections of PCB concentrations in the Chinook salmon-southern resident killer whale food web from the Northeastern Pacific. Simulations of scenarios are shown for (**A**) phytoplankton, (**B**) zooplankton, (**C**) foragefish (i.e. functional group that includes Pacific sardine, *Sardinops sagax caeruleus*; California/northern anchovy, *Engraulis mordax*; Pacific herring, *Clupea pallasi*; sand lance, *Ammodytes hexapterus*; eulachon, *Thaleichthys pacificus*; American shad, *Alosa sapidissima*; surf smelt, *Hypomesus pretiosus*; whitebait smelt, *Allosmerus elongates*), (**D**) squid, (**E**) Chinook salmon and (**F**) southern resident killer whales. Simulations were conducted with the EwE model and Ecotracer module under RCP 2.6 and 8.5 scenarios, and the reference baseline scenario (no climate change forcing control: black dashed line).

**Figure 4:**
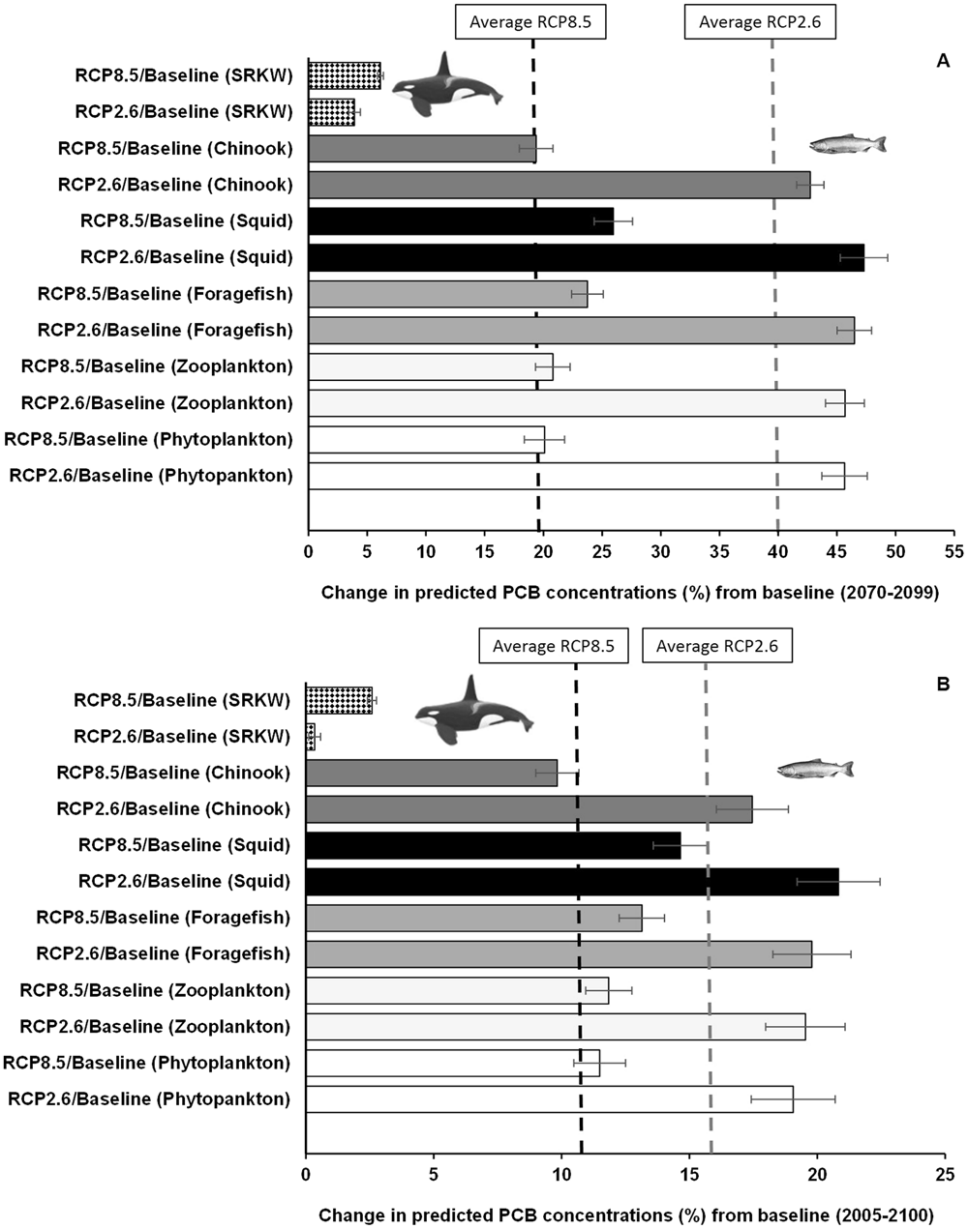
Mean percentage change in PCB concentrations projected in the food web from 2070 to 2099 (**A**) and for the entire simulation period (2005–2099). (**B**) Bars represent the mean ± 95% CI of changes under RCP 2.6 and RCP 8.5 scenarios relative to the reference baseline (i.e. no-climate change control) for phytoplankton, zooplankton, foragefish, squid, Chinook salmon and southern resident killer whales (SRKW). The black and grey dashed lines represent the average PCB concentrations from 2070–2099 and from 2005–2099 under the ‘business-as-usual’ (RCP 8.5) and ‘strong mitigation’ (RCP 2.6) scenarios, respectively.

In contrast to MeHg, although PCB concentrations were also projected to be amplified by climate change, the level of amplification was lower under the higher emission RCP 8.5 scenario (Fig. 3A–F). Average PCB concentrations from 2070–2099 under the ‘strong mitigation’ (RCP 2.6) and ‘business-as-usual’ (RCP 8.5) scenarios were 40 and 20%, respectively, higher for all functional groups relative to the reference scenario (Fig. 4A). However, except for killer whales, amplifications of bioaccumulation of PCBs in most other species groups were projected to be lower by 2090 under RCP 8.5 relative to RCP 2.6. For instance, from 2005–2099, the average (±95% CI) percent increases in PCBs under each scenario were 17.5% (±1.41%) and 10% (±0.84%) in Chinook salmon under RCP 2.6 an RCP 8.5 (Table 1), respectively. In contrast, PCBs concentration in SRKW increases by 0.35% (±0.23%) and 3.0% (±0.17%) in SRKW under the low and high emission scenarios, respectively (Fig. 4B and Table 1).

All functional groups of species have similar efficiency of MeHg bioaccumulation under RCP 8.5 relative to RCP 2.6 (Fig. 1). Specifically, the significant contribution of MeHg of squid under RCP 8.5 is further compensated by the increased bioaccumulation of other functional groups (Figs 1 and 2) serving as prey items for Chinook salmon (Fig. S6).

While projected PCB concentrations in phytoplankton, zooplankton, forage fish, and squid displayed large temporal variations under long-term climate signals (RCP 2.6 and RCP 8.5) with positive trends towards the end of the century under changes in sea surface temperature and net primary production (Supplementary Figs S4 and S5; Supplementary Information), fluctuation in PCB concentrations were not projected (i.e. steady state) under the no climate change scenario, i.e. reference baseline (Fig. 3A–D). Conversely, Chinook salmon (Fig. 3E) exhibited an initial, moderate period of decreasing concentrations (i.e. over 2005–2010) followed by a slight increasing trend in PCB concentrations after 2050. Such non-linear trend is driven by an initial decrease in dietary intake of more PCB-contaminated forage fish and squid, both of which also exhibit relatively higher PCB concentrations over the 2050–2010 period (Fig. 3C,D). In the long term, the simulations show increases in squid biomass (Fig. S6), and thus, in prey for Chinook salmon (i.e. the % of squid as prey increased from ~10% in 2005 to >20–30% by 2100 under RCP 8.5) although the availability of forage fish continue to decrease (as % of prey, forage fish decreased from 33% in 2005 to >20–30% by 2090–2100 under RCP 8.5; Fig. S6). The percent of prey consumed by Chinook salmon under RCP 2.6 is relatively constant over the simulation period (Fig. S7) in comparison to the prey proportion under RCP 8.5 (Fig. S6).

Similar to Chinook salmon, the contribution of Chinook salmon as the major prey item of SRWK, through which most of PCBs and MeHg intake is from, is projected to decrease from 90% in 2005 to 86.7% by 2100 under RCP 2.6; however, Chinook salmon remains relatively constant as a prey (~90%) under RCP 8.5. For the secondary prey of SRKWs, chum salmon (*O. keta*) increases from 5% in 2005 for both RCP 2.6 and RCP 8.5 to 8% and 9.5% in 2100 for RCP 2.6 and 8.5, respectively. Conversely, Coho salmon (*O. kisutch*) decreases from 5% in 2005 to 1.0% by 2100 under RCP 8.5. These differences in prey percent can be illustrated in terms of the RCP 8.5/RCP 2.6 ratio of prey proportion for SRKW, as depicted in Fig. S8. As an example, Fig. S8 (Supporting Information) shows that the RCP 8.5/RCP 2.6 ratio for Coho salmon dwindles from 1.0 at the initial time of simulation to 0.3 by 2100 (i.e. 70% reduction).

**Figure 5:**
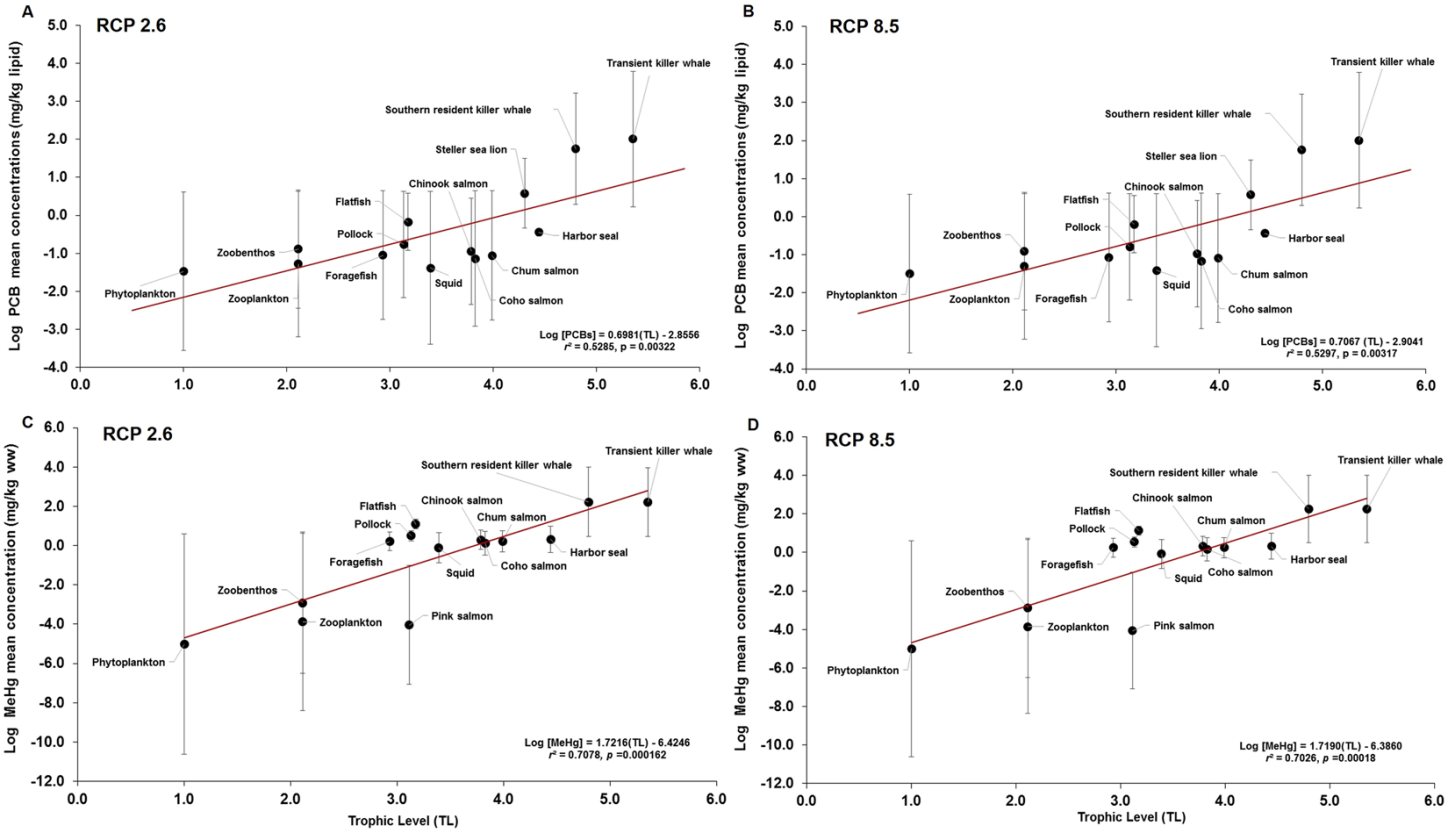
Relationships between the logarithm (log_10_) of projected contaminant concentrations, simulated under RCP 2.6 and RCP 8.5 scenarios, and trophic level (TL) throughout the entire simulation period in the marine food web. (**A**) log PCBs vs TL under RCP 2.6; (**B**) log PCBs vs TL under RCP 8.5; (**C**) log MeHg vs TL under RCP 2.6; and, (**D**) log MeHg vs TL under RCP 8.5. Data points (solid circles) represent the overall log average ± log SD (error bars) concentrations calculated for the entire simulation period (2005–2100). For reference purposes, PCB data predicted for harbor seals (*Phoca vitulina*), Steller sea lions (*Eumetopias jubatus*), and transient killer whales (*O*. *orca*) are also included as reference data points. The apparent trophic magnification factor (TMF) was calculated from the slopes of the relationships (see Table S6 in Supplementary Information).

The trophic magnification factor (TMF; see Methods) was calculated as metric of biomagnification for PCBs and MeHg in the food web for the entire simulation period subject to climate change forcing. A significant increase in predicted log-PCB and log-MeHg concentrations correlated with trophic level for both RCP 2.6 and RCP 8.5 scenarios (Fig. 5A–D) was predicted. The TMF of PCB and MeHg under RCP 2.6, RCP 8.5 and the no climate change scenarios was significantly greater than one (TMF > 1), i.e. 4.99–5.30 for PCBs; and, 52.4–53.1 for MeHg across the climate change scenarios (Supplementary Table S8).

While the TMF values for MeHg were higher than those for PCBs (i.e. average MeHg-TMF/PCBs-TMF ratio ≈ 10), TMFs were basically similar under the RCP 2.6 and RCP 8.5 scenarios as well as under the no climate change scenario for either PCBs or MeHg, as shown in Table S8. These findings suggest the lack of attributable effects by climate change on TMFs in the simulations for this particular food web despite the increasing changes projected across most species groups, under the influence climate change forcing (Figs 2 and 4).

**Figure 6:**
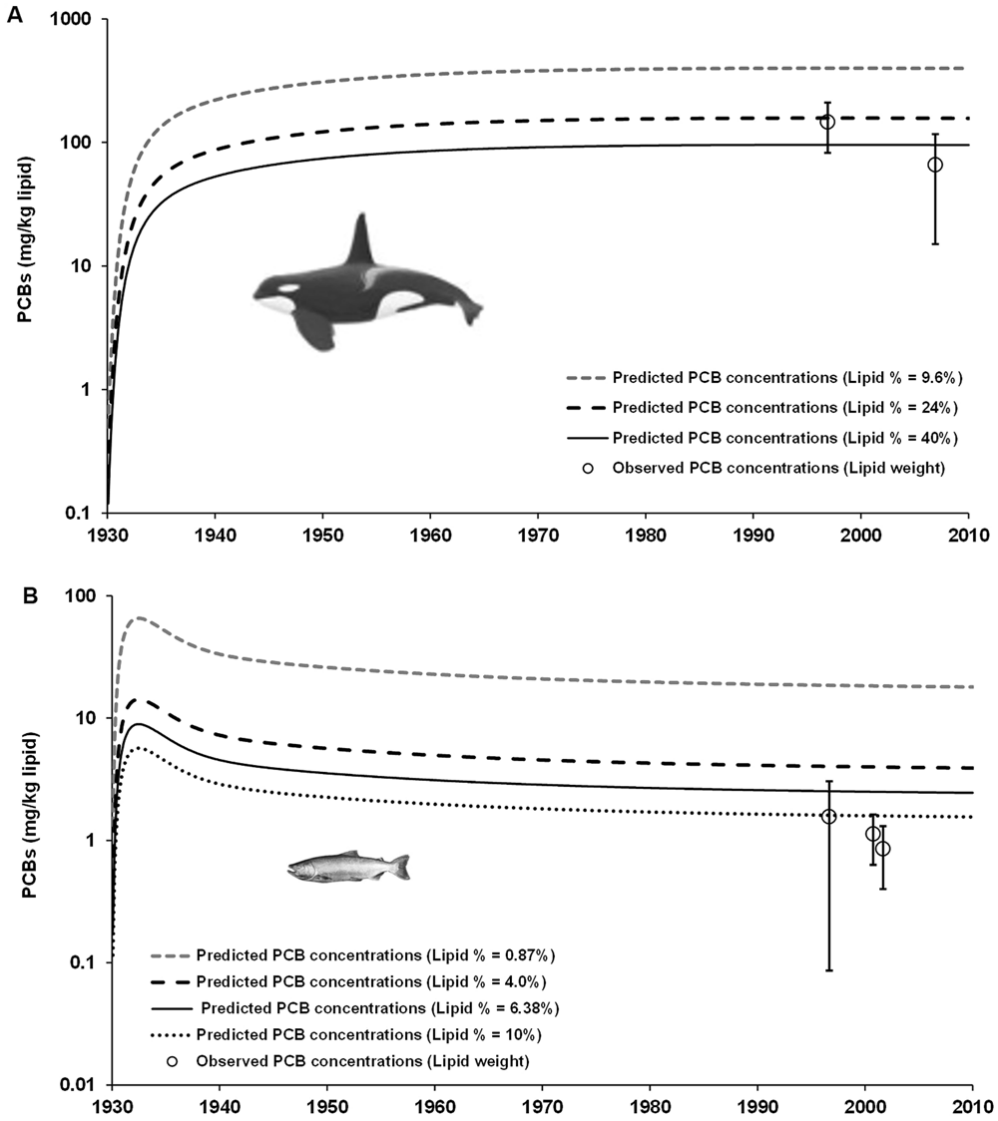
Historical projections of PCB concentrations (mg/kg lipid) in southern resident killer whales (SRKW) (**A**) and Chinook salmon (**B**) simulated with the Ecotracer routine of the EwE model and adjusted to different values of lipid content (%). Projected PCB concentrations of PCBs lipid normalized to relatively normal values of lipid content reproduce concentrations close to empirical data observed in both species, as reported elsewhere^18–21^. The open circles are the average PCB concentration observed in SRKW and Chinook salmon and error bars are 95% CI. By normalizing the PCB concentrations to low lipid fractions observed in killer whales (i.e. lipid content = 9.6%) and Chinook salmons (i.e. lipid content = 0.87%), the concentrations of PCBs are amplified in these species as an indication of a bioamplification process.

**Figure 7:**
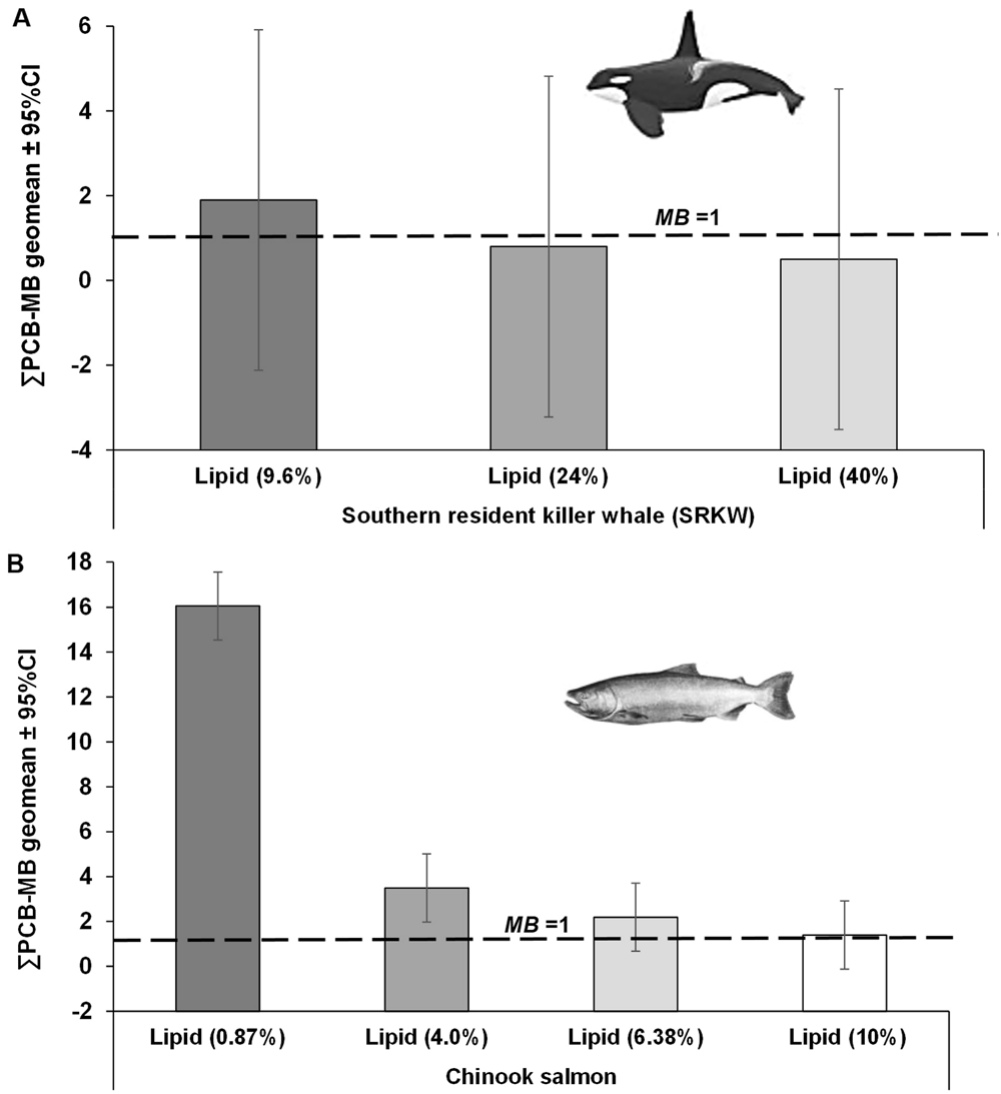
Geometric mean of the model bias (*MB*) of total PCBs (ΣPCB) for southern resident killer whale (SRKW) (**A**) and Chinook salmon (**B**). The error bars are 95% confidence intervals as indication of the uncertainty of model predictions. Lipid content for SRKW and Chinook salmon were retrieved from data reported elsewhere^18–21^. Dashed line means *MB* = 1.0 (i.e., equal concentration values when comparing projected to observed data).

Outcomes of the model bias (*MB*) evaluation are shown in Figs 6 and 7 and Tables S5 and S6. The *MB* analysis showed that the projections of historical PCB concentrations in Chinook salmon and SRKW male resulting from the Ecotracer simulation reproduced fairly well the recent empirical PCB data observed in these two species from the Northeastern Pacific^18–21^, as illustrated in Fig. 6A,B. Depending on the PCB concentrations and normal ranges of lipid percent reported for these species (i.e. ranging 9.6–40% for SRKW, Fig. 7A; and, 0.87–10% for Chinook salmon, Fig. 7B), the model bias (*MB* ± *MB*_SD_) showed little underprediction (i.e. 0.5 ± 2.9 or 0.8 ± 2.9) or/and overprediction (i.e. 1.9 ± 2.9) for SRKW (Fig. 7A and Table S5), while for Chinook salmon there was overprediction (i.e. *MB* ± *MB*_SD_ = 3.5 ± 1.34 or 16.0 ± 1.34 at low lipid contents of 4% and 0.87%, respectively), as indication of bioamplification due to low lipid reserves (Fig. 7B and Table S6). However, the *MB* show little overprediction (i.e. 1.4 ± 1.34) with a lipid content of 10% in Chinook salmon.

## Discussion

Our simulations show the expected differences between MeHg and PCBs due to pollutant amplification under climate change. These differences are driven by different dietary uptake and total elimination rates and species biomass transfer through the food web. These differences are also expected to occur because the biotransformation and biomagnification of pollutants, and bioenergetics in ectothermic and endothermic species vary greatly, i.e. endothermic animals more likely to biomagnify chemicals than ectothermic species^22,23^. The specific bioaccumulation and thermodynamics of organic pollutants also diverge between gill-ventilating/water-respiring organisms and air-breathing organisms, responding differently to chemical bioaccumulation^22,24^ and temperature exposures^25^. Ultimately, changes in food web structure and metabolic efficiency (i.e. ratio of consumption to production in the food web) leading to changes of biomass in functional groups of species of the food web are mechanisms influencing PCB and MeHg bioaccumulation and biomagnification under climate change forcing.

The discrepancies in amplification patterns of bioaccumulation between pollutants and species to climate change are likely to be explained in part by differences in the bioaccumulation rate of pollutants (i.e. pollutant-specific bioaccumulation) and the sensitivity to climate effects between functional groups (i.e. species-specific bioaccumulation) (Fig. 4A,B). Changes in relative abundance of prey and predator functional groups affect the pathways of bioaccumulation. For example, squid, an important prey item for Chinook salmon, and other species (e.g., hake, *Merluccius productus;* rockfish, *Sebastes* spp.; pink salmon, *O*. *gorbuscha*) are higher in biomass projected under RCP 8.5 relative to those under RCP 2.6 (i.e. biomass ratio RCP 8.5 to RCP 2.6 >1; Fig. S9). In contrast, other functional species (i.e. forage fish, flatfish) are projected to have relatively lower biomass under RCP 8.5 than under RCP 2.6 (Fig. S9). As a result, squid (trophic levels, TL = 3.4) becomes a more important source of biomass to support the production of predatory species such as salmon (TL = 3.9) and killer whales (Tl = 4.8) under RCP 8.5. As the bioaccumulation of PCB is more efficient through squid than other prey items of Pacific salmon (i.e. Chinook salmon), the concentration of PCB in these predatory species is higher under RCP 2.6 (i.e. the lower biomass of squid under RCP 2.6 increases its per unit PCB concentration).

While there is a food web-specific pollutant biomagnification process driving the long-term bioaccumulation pattern for either MeHg or PCBs in SRKWs, the high trophic level, long life span, and less pulse in the declining biomass of SRKWs in response to both climate change forcing and contaminant burden in major prey (Pacific salmon, i.e. Chinook salmon) are plausible reasons to explain in part the behavior of these contaminant trends in SRKWs over the simulation period.

The increase in projected MeHg concentrations observed in salmon and SRKW can be explained by the continuous accumulation of MeHg through diet combined with a slow elimination and also a long biological half-life in these animals^26,27^. Mercury concentrations in large predatory fish and cetaceans are strongly influenced by the length of and changes in structure of the food web^6,17,22,27^. Moreover, low trophic level organisms from coastal-marine food webs can play a crucial role in the trophic transfer of MeHg, as climate change can increase its exposure and enhance the MeHg bioaccumulation factor in zooplankton by a factor of 2 to 7, as reported by Jonsson *et al*.^7^. Here, we demonstrated that bioaccumulation in zooplankton increases by 3% under RCP 2.6 and by 12% under RCP 8.5 relative to the reference baseline (control) scenario (i.e. no climate change) for the simulation period 2005–2099 (Table 1 and Fig. 2B). The increase in MeHg concentration in zooplankton is much higher (i.e. 9.5% for RCP 2.6 and 22% for RCP 8.5) at the end of the century (i.e. simulation period 2070–2099; see Fig. 2A).

The projected decrease in simulated PCB concentrations in SRKW is similar to past trends observed in recent modelling work not including climate change forcing^28^. PCB concentrations in the northeast Pacific declined rapidly in most marine fauna following the PCB-ban in the 1970s. The decrease in POPs under global control by the Stockholm Convention should be expected in marine biota^2,28^. However, the metabolic recalcitrance of PCBs can delay responses to such declines in large, long-lived species, including the highly PCB-contaminated SRKW of the Northeastern Pacific^1,28^. Particularly, PCB contamination in this killer whale ecotype, specialized in consuming salmon, can be further exacerbated during periods of nutritional stress and bioenergetic requirements when the abundance of highly PCB-contaminated Chinook salmon is the lowest during ocean warming conditions (i.e. climate change-induced contaminant sensitivity)^1,29^. This is consistent with our model outputs that show bioamplification of PCBs, increased by one order of magnitude, when lipid reserves are the lowest in both Chinook salmon and SRKWs, as shown in Fig. 6.

Decreasing biomass of key species groups in terms of energy flow and protracted reduction and density of apex predators under RCP 2.6 and RCP 8.5 at the end of the century (Fig. S9) is a possible contributing factor influencing the lack of difference in TMFs when compared against the reference scenario (no climate change). We postulate that pollutant bioamplification could reach a climax level due to impending contaminant oversaturation across the food web under low or high CO_2_ emissions (i.e. RCP 2.6 or RCP 8.5), at which additional trophic magnification of contaminants cannot be detected (i.e., the food web and the ecosystem could be inhibited and unable to continue bioamplifying contaminants).

Simulations of the influence of dynamic temperature scenarios (e.g., high contaminant concentrations) compared to constant temperature scenarios on the body weight and contaminant concentration in fish and seabirds have demonstrated the bioamplification process^25^. Pollutant bioamplification is a special case of bioaccumulation, driving increasing concentrations of organic contaminants when marine animals lose weight during periods of nutritional stress, fasting, reproduction and migrations^25^. In the context of climate change, the shrinking of fish due to the alteration of the metabolic scope rate caused by ocean warming, reduced oxygen and other biochemical factors^30^ can contribute to pollutant bioamplification^1^.

Some assumptions were required for simplicity due to data limitations. For instance, the octanol-water (*K*_*OW*_) and octanol-air (*K*_*O*A_) partition/distribution coefficients for PCBs and MeHg under ambient/ectotherm and endotherm temperatures are not directly accounted for in the model; excretion of PCBs and MeHg was assumed to be negligible for most organisms because of high uptake and absorption of PCBs and MeHg (i.e. we assumed that 100% of PCBs and MeHg is absorbed for the purpose of this work) in marine biota in general (see Tables S4 and S5 for input data and references). MeHg biogeochemistry was not directly accounted in the model. Water column and sediment MeHg concentrations can be changed by SST, pH and dissolved oxygen (DO) concentration (e.g., photodegradation rate formation/reductions rates of MeHg)^1,4,7^. Particularly formation and degradation rates of MeHg in seawater are largely DO, pH, UV availability, and dissolved organic carbon (DOC)^4,7^. Future studies could incorporate direct effects of changing ocean biogeochemistry on contaminants bio-availability into the model.

The outcomes of the model bias analysis generally agrees with predicted PCB concentrations in SRKW from 1930 forward to the early 2000s reported by Hickie *et al*.^28^.

As empirical data for mercury concentrations (MeHg) are limited for the majority of marine organisms of the food web from the study region (e.g., absence of MeHg data for killer whales precluded the *MB*-assessment for this species), we conducted a comparison (i.e. *MB* ratio) of projected MeHg concentrations for wild Chinook, chum and coho salmon species against the observed MeHg data reported for these species in coastal BC in 2003^31^. Therefore, based on the limited empirical MeHg data reported for these salmon species (i.e. Chinook, chum and coho) for a single year^31^, the model simulations showed systematically overprediction when compared to observed MeHg concentrations (Fig. S10 and Table S7). The lack of empirical MeHg data for most species/functional groups prevented a concerted assessment of the model bias and uncertainty. However, the accuracy of the simulations for the model was tested by comparing the model PCB projections in biota to empirical data and calculating 95% CI around the geometric mean of the *MB*, as an indication of the uncertainty of model predictions (Figs 6 and 7; Tables S5 and S6).

Several empirical studies in the Arctic provide evidence of the influence of climate change in increasing concentrations of POPs in polar bears^6^ and mercury in sea birds^17^. Conversely, some field contributions have shown that concentrations of many legacy POPs have decreased in food webs of seabirds or exhibited a lack of increase in polar bear populations^6^. While MeHg concentrations are increasing, the decrease in POPs has been attributed to shifts in diets and in trophic position, and the phase-out of some POPs (i.e., PCBs, DDTs) in the late 1970s^6,28^. The model projections for MeHg are also consistent with the outcomes of a climate change-mercury bioaccumulation EwE-model, involving Atlantic cod (*Gadus morhua*) and pilot whales (*Globicephala melas*)^26^.

Our findings reveal that climate change and contaminants can potentially combine to trigger pollutant bioamplification in the food web, improving our understanding of climate-pollution interactions. A more comprehensive awareness of the risks of multiple anthropogenic stressors can thus highlight key areas for concerted research and potential pollution mitigation policies including limiting mercury and greenhouse gas emissions from human activities.

## Methods

### Study Area

We explore and focus our analyses and projections on a marine food web of the Salish Sea Ecosystem (i.e. the Strait of Georgia, British Columbia (BC), Canada, and Puget Sound, WA, USA) and offshore waters in the Northeastern Pacific Ocean, mainly focused on the Canada’s Pacific marine ecosystems (see Supplementary Fig. S1).

### Food web

The Chinook salmon-southern resident killer whale food web of the Northeastern Pacific has several functional groups or species, including phytoplankton, three invertebrate groups, including zoobenthos, zooplankton and squids, fish groups, and marine mammal groups represented within an Ecopath with Ecosism (EwE) ecosystem model (see Supplementary Fig. S2 and Table S1, Supplementary References). The diet composition matrix for species and functional groups represents the ecosystem’s food web, including apex marine predators such as resident and transient killer whales (see Supplementary Information).

### EwE with Ecotracer module

We use the Ecopath with Ecosism model (EwE), a tropodynamic ecosystem approach to simulate the flow of mass and energy across food webs^32^ (see Supplementary Information and supplementary references). The EwE Ecotracer module simulates contaminant bioaccumulation in the food web (see Supplementary Information and supplementary references). The linear dynamical equation for time changes in contaminant concentration (C*i*B*i*/dt) in a given species group *i* is represented as follows^32^:

1 $$\begin{array}{c}{\rm{C}}i{\rm{B}}i/{\rm{dt}}=(Cj\,\bullet \,{\rm{GC}}i\,\bullet \,Qji/Bj)+({\rm{u}}i\,\bullet \,Bi\,\bullet \,Co)+(ci\,\bullet \,Ii)-[({\rm{C}}i\,\bullet \,{\rm{Q}}ij/{\rm{B}}i)\\ \,\,\,\,\,+{\rm{C}}i\,\bullet \,MOi+((1-{\rm{GC}}i)\,\bullet \,\sum jCj\,\bullet \,Qji/Bj+ei\,\bullet \,Ci+di\,\bullet \,Ci)\end{array}$$

The description of the equation terms is as follows:

1. Uptake from food: *Cj* • *GCi* • *Qji/Bj* where *Cj* = conc in food j, *GCi* = proportion of food assimilated by type *i* organisms; *Qji* = biomass flow rate from *j* to *i* (estimated in Ecopath as *Bi* • (*Q/B*)*I* • *DCij*) *i*, *Bj* = food j biomass;
2. Direct uptake from environment: *ui* • *Bi* • *Co*, where *ui* = parameter representing uptake per biomass per time, per unit environmental concentration, *Bi* = biomass, Co = environmental concentration;
3. Concentration in immigrating organisms: *ci* • *Ii*, where *ci* = parameter (tracer per unit biomass in immigrating biomass), *Ii* = biomass of pool i immigrants per time;
4. Predation: *Ci* • *Qij/Bi*, where *Ci* = concentration in pool *i*, *Qij* = consumption rate of type *i* organisms by predator type j, *Bi* = biomass in pool i;
5. Detritus: *Ci* • *MOi* + (1 − *GCi*) • *∑jCj* • *Qji*/*Bj*, where *MOi* = non-predation death rate of type *i* (per year), *GCi* = fraction of food intake assimilated, *Qji* = intake rate if type *j* biomass by type *i*;
6. Emigration: *ei* • *Ci*, where *ei* = emigration rate (per year);
7. Metabolism: *di* • *Ci*, where *di* = metabolism + decay rate for the material while in pool *i*.

### Climate Predictions and Simulations

To predict changes of the four climate change factors used to drive the EwE and Ecotracer modelling approach, we used climate change data from the Earth System Model using Modular Ocean Model ESM2M developed by the NOAA- Geophysical Fluid Dynamics Laboratory (GFDL)^33^, combined with representative concentration pathways (RCPs), including RCP 2.6, which was used as an optimistic climate scenario, and RCP 8.5 as the pessimistic scenario (Fig. S3). Because there are no downscaled predictions for the study area, we used the results predicted for a larger region (Northeastern Pacific), engulfing the Hecate Strait, Queen Charlotte Sound, Strait of Georgia and Puget Sound, as well as outer coast of BC and offshore waters of Haida Gwaii and Vancouver Island’s west coasts (Fig. S1). Thus, predictions for temperature, pH, oxygen and primary production (chlorophyll a) were obtained from ESM2M (over the period 1950–2100 for RCP 2.6 and RCP 8.5), as shown in Appendix I.

Based on Guenette *et al*.^34^, we assumed that all physical-chemical factors (temperature, pH and oxygen) affected the scope of growth of species and vulnerability of prey. This was modelled in Ecopath with Ecosim by introducing a forcing function (time series of relative change according to the climate predictions) that influences the ratio of consumption to biomass (Q/B) of each species by directly modifying the prey vulnerability for predators in the EwE model^34,35^. This assumes that the predicted changes in environment will make the species a more or less efficient predator, converting consumers into super predators or inferior predators, and resulting in increased/decreased growth and thus, increasing or decreasing their biological productivity^34,35^. Changes in primary production were modelled using a forcing function directly on primary production, modifying biological production^34,35^.

The time period baseline for climate change forcing factor data used in the model represents the average state by 2005 (i.e. 20 years average from 1996 and 2015). Then, the time series data for each climate change factor in a given year were adjusted to this average to produce an index (rescaled to 1). Thus, the predicted effects of climatic factors on growth and prey vulnerability of the predator were at the last year of the fitted EwE model (2015). The simulations described here begin at the end of the fitted period (2015) and were run until 2099.

The simulation scenarios (RCP 2.6 and RCP 8.5) were compared to a baseline reference scenario in the absence of climate change forcing, simulated in the EwE model with Ecotracer.

### PCB and MeHg Data

Empirical contaminant data for PCBs and MeHg was obtained from the existing literature^18–21,31,36–38^ for the study area, as documented elsewhere (see Supplementary Information).

### Apparent Trophic Magnification Factor

The trophic magnification factor (TMF) is defined as the change in log-concentration of a chemical per trophic level in a food web and often used to explore the biomagnification of pollutants in an entire food-web^39^ (see Supplementary Information). The TMF was determined for the simulations (i.e. 2005–2100) of PCBs and MeHg concentrations under RCP 2.6, RCP 8.5 and no climate change scenario, and calculated as the antilog of the regression slope (i.e. TMF = 10^*b*^, where *b* is the slope) of the linear regression between the log transformed concentrations of the contaminant (i.e. PCBs, MeHg) predicted in organisms of the food web and their trophic level (TL)^39^:

2 $$\mathrm{log}\,[{\rm{PCBs}}]=a+b\,({\rm{TL}})$$

3 $$\mathrm{log}\,[\mathrm{MeHg}]=a+b\,({\rm{TL}})$$

More details on the calculation of the TMF in Supplementary Information; regression statistics results are reported in Table S8.

### Model Bias and Performance

As a prerequisite to test and compare the projections of contaminants under the two climate scenarios, we first build confidence that our simulation scenarios under RCP 2.6 and 8.5 provide a reasonable representation of PCBs (used here as the standard reference contaminant for which empirical data in biota are readily available in the study region) in selected marine biota, for which empirical data are available in the study region. To quantitatively express model performance for total PCBs (ΣPCBs), we used the model bias *MB*^36,37^ (see Supplementary Information):

4 $$M{B}_{i}={10}^{\sum _{i=1}^{n}\frac{[\mathrm{log}({C}_{BP,i\Sigma PCB}/{C}_{BO,i\Sigma PCB})]}{n}}$$

where *C*_BP,*i* ΣPCB_ and *C*_BO,*i* ΣPCB_ are, respectively, the model calculated and observed ∑PCB concentrations in each species *i* for observations *n* ranging from *n* = 1 to the total number of concentration measurements. Outcomes of the *MB* evaluation are shown in Figs 6 and 7 and Tables S5 and S6 for PCBs, and in Fig. S10 and Table S7 for MeHg.

## Electronic supplementary material

Supplementary Information
