# Supplementary material for: Projected amplification of food web bioaccumulation of MeHg and PCBs under climate change in the Northeastern Pacific
Source: Sci Rep. 2018 Sep 7;8:13460. doi: 10.1038/s41598-018-31824-5 (PMC6128847; doi:10.1038/s41598-018-31824-5)
Supplement: Supplementary file 1 — Supplementary Information [file 41598_2018_31824_MOESM1_ESM.docx]

**Supplementary Information**

**Projected amplification of food web bioaccumulation of MeHg and PCBs under climate change in the Northeastern Pacific**

Juan José Alava^1*^, Andrés M. Cisneros-Montemayor^1^, U. Rashid Sumaila^1^, William W.L. Cheung^1^

^1^Institute for the Oceans and Fisheries, University of British Columbia, 2202 Main Mall Vancouver, BC V6T 1Z4, Canada. *E-mail: [j.alava@oceans.ubc.ca](mailto:j.alava@oceans.ubc.ca)

**Study Area**

We explore and focus our analyses of the interplay of climate change and chemical pollutants impacts on a marine food web of the of the Northeastern Pacific Ocean, including the inshore and coastal waters of Salish Sea Ecosystem (i.e. the Strait of Georgia, British Columbia, Canada, and Puget Sound, WA, USA), Queen Charlotte Sound and Hecate Strait in Canada west coast, as well as offshore waters located to the west coasts of Haida Gwaii and Vancouver Island (BC, Canada). Thus, the spatial coverage is mainly focused on the Canada’s Pacific marine ecosystems (as shown in Fig. S1), using and adapting an ecosystem model for the Northeastern Pacific^1^. The model offers almost continuous spatial coverage of our region of interest influenced by the Alaskan and Northern California Currents^1^.

**Figure S1**. Location of the study area on the Northeastern Pacific. The polygon demarcated by thick red lines shows the area coverage by the data from Earth System Model using Modular Ocean Model (ESM2M), NOAA- Geophysical Fluid Dynamics Laboratory (GFDL) (Dunne et al., 2012), used for the development of simulations under climate change forcing. The geographical coordinates used to demark the polygon were set up at four reference points located at about 54°23'26.39"N, 129°20'33.65"W; 48° 6'2.14"N, 121°22'23.09"W; 47°43'46.23"N, 130°35'24.54"W; and, 54° 0'12.77"N, 138° 3'12.99"W. The map was created using Google Earth Pro software and is used for this journal with permission following the terms of service and attribution available at <https://www.google.com/permissions/geoguidelines.html> . The attribution along with copyright notices are depicted in the lines shown on the bottom (center) of the map content (Data LDEO-Columbia, NSF, NOAA© 2016 Google, Data SIO, NOAA, U.S. Navy, NGA, GEBCO, Image Landsat/Copernicus).

**Marine food web representation and structure**

The food web for the Salish Sea ecosystem has 20 functional groups or species, including phytoplankton as a primary producer group, three invertebrates groups, including zoobenthos, zooplankton and squids, ten fish groups, and six marine mammal groups (Fig. S2; Table S1). The diet composition matrix for species and functional groups basically represents the food webs of the apex marine predators, including resident and transient killer whales, of the Salish Sea Ecosystem. Detritus as a food source for some species groups is also included as basic input in the diet matrix. While several functional groups and species are included in the trophodynamic ecosystem model, the modelling work is mainly focused on the Chinook salmon-southern resident killer whale food web (Fig. S2). The food-web for resident killer whales as a demonstrative food-web was used for the model because data for representative species are available^2-4^. The food web model is based on detailed studies that confirm that resident killer whales (*O. orca*) are fish-eating marine mammals with a strong preference for Chinook salmon (*Oncorhynchus tshawytscha*), the largest species of Pacific salmon (i.e.≈50 kg) in the Pacific Northwest, accounting for 72% of the resident killer whales' diet^5-7^. Other fish species in the resident killer whales' diet are halibut (*H. stenolepis*), sablefish (*A. fimbria*), lingcod (*Ophiodon elongates*) and several other species of Pacific salmon, including pink (*Oncorhynchus gorbuscha*), coho (*O. kisutch*) and chum (*O. keta*) salmon ^5,7^. Chinook salmon spend the majority of their life in the open ocean (i.e. five years as oceanic adult stage), where they feed on foragefish such as Pacific herrings (*Clupea pallasii*) and squid ^2,8^.


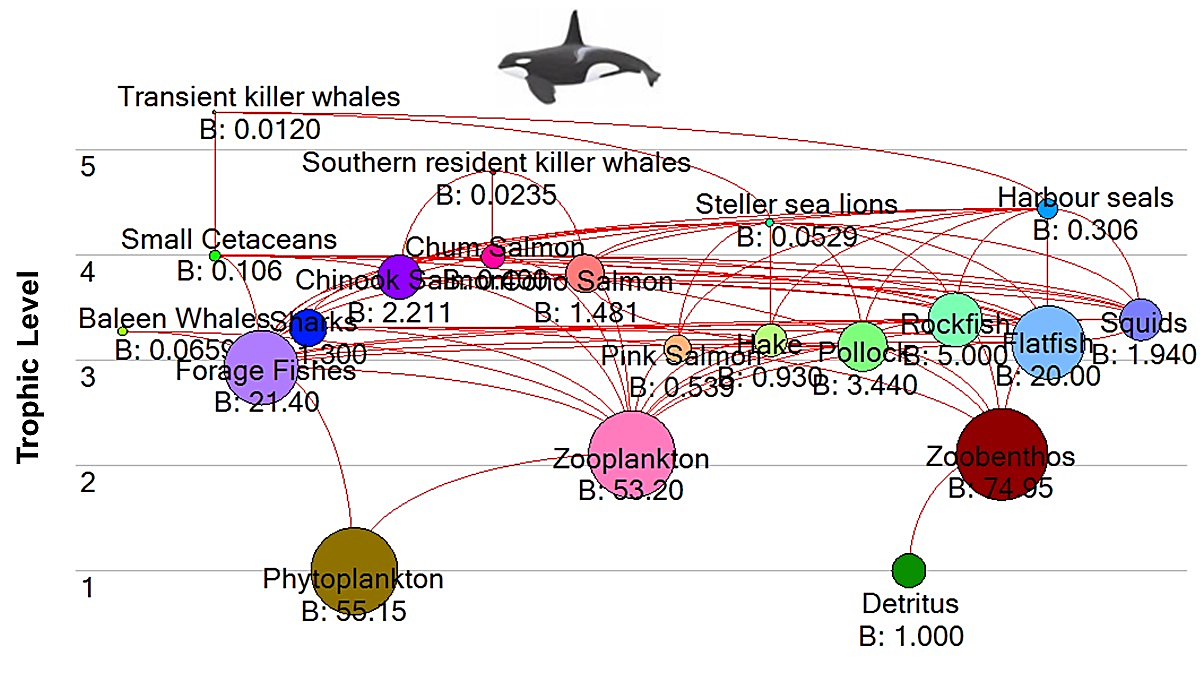
**Figure S2.** Schematic illustration of the marine food web of the Northeastern Pacific (i.e. Chinook salmon-southern resident killer whale food web) used in the EwE model. The trophic level or average trophic level for each species/taxon or functional group, as well as the biomass (B in tonnes/km^2^) initially used for each functional group are also shown. The size of the filled circles for each species of functional group is proportional to the biomass density available in the marine ecosystem and food wed.

**Table S1**. Species and functional groups of the marine food web included in the EwE model.

| Specific species or/and functional groups | Common names/Scientific names of representative species per functional group |
| --- | --- |
| Transient killer whales | Transient or Bigg’s killer whales (*Orcinus orca*) |
| Resident killer whales | Southern resident killer whale (*Orcinus orca*) |
| Small toothed cetaceans | Pacific white-sided dolphin (*Lagenorhynchus obliquidens*); harbour porpoise (*Phocoena phocoena*); Dall's porpoise (*Phocoenoides dalli*); Risso's dolphin (*Grampus griseus)*; northern rigth whale dolphin (*Lissodelphis borealis*) |
| Baleen whales | Humpback whale (*Megaptera novaeangliae*); minke whale (*Balaenoptera acutorostrata*); fin whale (*B. physalus*); sei whale (*B. borealis*); blue whale (*B.* *musculus*); Gray whale (*Eschrichtius robustus*) |
| Sea lions | Steller sea lion (*Eumetopias jubatus*); California sea lion (*Zalophus californianus*) |
| Seals | Harbour seal (*Phoca vitulina*) |
| Sharks | Spiny dogfish (*Squalus acanthias*); cat sharks (*Apristurus* spp.); thresher sharks (*Alopias* spp.); blue sharks (*Prionace glauca*); soupfin sharks (*Galeorhinus galeus*); salmon shark (*Lamma ditropis*); basking sharks (*Cetorhinus maximus*); Pacific sleeper sharks (*Somniosus pacificus*); great white shark (*Carcharodon carcharias*) |
| Chinook Salmon | *Oncorhynchus tshawytscha* |
| Chum Salmon | *Oncorhynchus keta* |
| Coho Salmon | *Oncorhynchus kisutch* |
| Pink Salmon | *Oncorhynchus gorbuscha* |
| Hake | *Merluccius productus* |
| Pollock | *Theragra chalcogramma* |
| Rockfish | Canary (*Sebastes pinniger*), yellowtail (*S. flavidus*), widow (*S. entomelas*), black (*S. melanops*), shelf rockfish (*Sebastes* spp.) , slope rockfish (Sebastes spp.), chilipepper (*S. goodei*), bocaccio (*S. paucispinis*) thornyheads (*Sebastolobus alascanus*) |
| Flatfish | Dover Sole (*Microstomus pacificus)*; English sole (*Parophys vetulus*); Petrale sole (*Eopsetta jordani*); Rex sole (*Glyptocephalus zachirus*); arrowtooth flounder (*Atherestes stomias*); small flatfish (i.e. Pacific sanddab, *Citharichthys sordidus/*spp.; slender sole, *Lyopsetta exilis;* sand sole*, Psettichtys melanostictus;* butter sole*, Isopsetta isolepis;* starry flunder*, Platichthys stellatus;* rock sole, *Lepidopsetta bilineata*); Pacific halibut (*Hippoglossus stenolepus*) |
| Forage Fishes | Pacific sardine (*Sardinops sagax caeruleus*); California/northern anchovy (*Engraulis mordax*); Pacific herring (*Clupea pallasi*); sand lance (*Ammodytes hexapterus*), eulachon (*Thaleichthys pacificus*), American shad (*Alosa sapidissima*), surf smelt (*Hypomesus pretiosus*), whitebait smelt (*Allosmerus elongates*) |
| Squids | Cephalopods, particularly California market squid (*Loligo opalescens*) |
| Zoobenthos  (Benthic infauna) | polychaetes, equinoderms, molluscs (bivalves), crustaceans (e.g. amphipods, Dungeness crab (*Metacarcinus magister*), Pandalid shrimps (*Pandalus borealis*), *Crangon* spp.,) |
| Zooplankton | Copepods, Euphausiids |
| Phytoplankton  Detritus | |

**Climate Predictions and Simulations**

Climate change data from the NOAA’s Geophysical Fluid Dynamics Laboratory Earth System Model 2M (GFDL ESM2M)^9^, combined with Representative Concentration Pathways (RCPs), including RCP 2.6 and RCP 8.5 were used to project changes of the four climate change factors to drive the EwE and Ecotracer model approach (see conceptual model framework of the modelling work in Fig. S3). Because there are no downscaled predictions for the study area, we used the results predicted for a larger region (Northeastern Pacific). Information on the climate change variables are reported in Supporting Information. Thus, predictions for temperature, pH, oxygen and primary production (chlorophyll a) were obtained from ESM2M (over the period 1950-2100; see Appendix I) to calculate climate change forcing functions (see Figs. S3 and S4). The simulation scenarios (RCP 2.6 and RCP 8.5) were compared to a baseline reference scenario (no-climate change) in the absence of climate change forcing, simulated in the EwE model with Ecotracer. Following Guenette *et al*.^10^, we assumed that all physical-chemical factors (i.e. temperature, pH and oxygen) affected the scope of growth of species and vulnerability of prey, influencing the ratio of consumption to biomass (Q/B) of each species by directly modifying the prey vulnerability for predators in the EwE model ^10,11^.


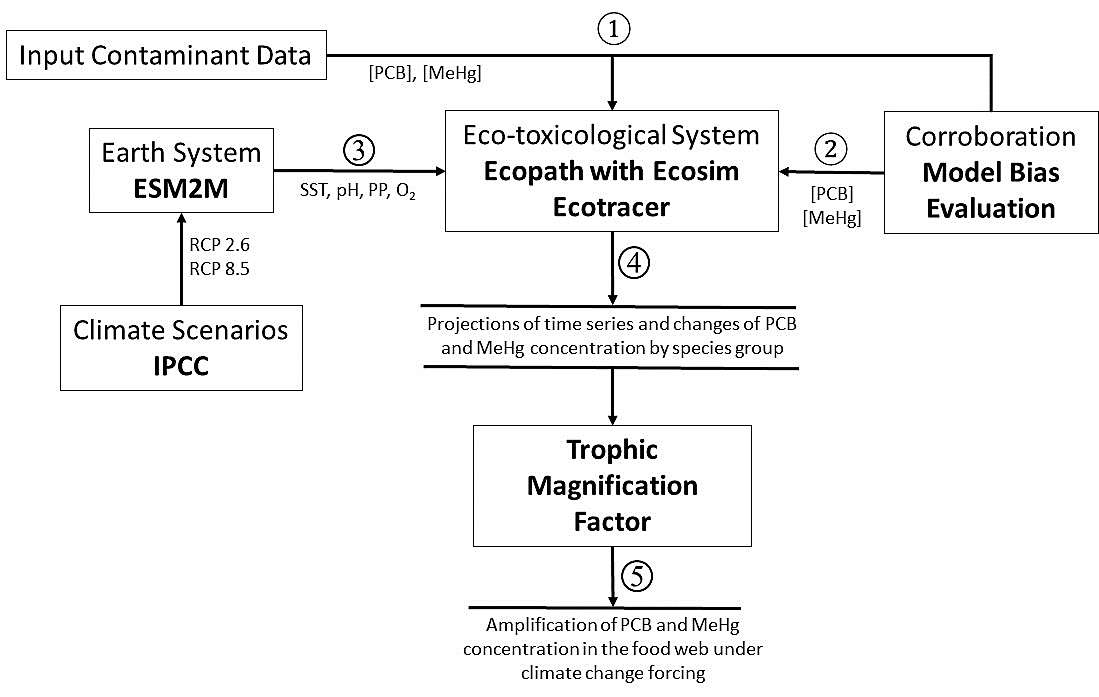


**Figure S3**. Conceptual model framework illustrating the basic relationships of modelling components to predict and assess the combined effect and interactions of climate change forcing and pollutants in marine food webs and ecosystems. **(1)** The empirical data for contaminants (PCBs, MeHg) is a key input for the modelling work to predict concentrations in a particular species or function group of species in marine food webs over time (the Ecotoxicological System: Ecotracer routine of the Ecopath with Ecosim (EwE) ecosystem model that simulates the bioaccumulation of a given contaminant in the food web); **(2)** to corroborate the projections resulting from the simulations, a model bias evaluation approach is required to assess the performance of the ecosystem model (i.e. whether the model is reproducing fairly well concentration values for PCBs and MeHg similar to those observed from the empirical contaminant data available). Then, **(3)** the climate change data is retrieved from the Earth System Model using Modular Ocean Model ESM2M (NOAA- Geophysical Fluid Dynamics Laboratory) in combination with the IPCC-representative concentrations pathways (RCP 2.6 and RCP 8.5) scenarios to produce climate change forcing functions (i.e. time series of relative change according to the climate predictions) for sea surface temperature (SST), pH, dissolved oxygen (O_2_), and primary production (PP), which are implemented as inputs in the EwE model. **(4)** Following the inclusion of climate change forcing functions (Ecosim) and contaminant data (Ecotracer), the Ecotoxicological System is run to simulate the concentrations in each species or functional group to produce the projections of time series of contaminants subject to climate change forcing in the food web. **(5)** The contaminant data generated is used to calculate trophic magnification factors (TMF) for PCBs and MeHg to confirm magnification or amplification of contaminants in the food web affected by climate change.

**Impact of climate change variables and data used for climate change forcing functions**

**Impacts of sea surface temperature (SST)**

In the Northeastern Pacific, both long term global warming and natural variability of ENSO events can contribute importantly to sea surface variability over decadal time scales, i.e. middle and long terms ^12,13^. Despite the long-term warming in Canada’s Pacific marine ecosystems, SST trends in the region have been anomalously cool since 2007 with La Niña-type conditions prevailing during the cool phase of the Pacific Decadal Oscillation, possibly masking future warming ^14^. However, the SST increased by about 1.0 °C in the Strait of Georgia and elsewhere around coastal British Columbia during the last century based on lighthouse records ^15-17^. For instance, recent measurements showed that the average daily SST was warmer by about 0.8 °C in 2015 compared to 2014 in coastal British Columbia ^17^.

As aforementioned, we retrieved predicted times series SST data from the ESM2M-GFDL^9^ in combination with RCP scenarios (RCP 2.6 and RCP 8.5) for our study region. This data served to calculate the temperature difference index (∆SST; Figs. S4A and S5A) to be incorporated in the calculations of the time series SST forcing function for each RCP scenario. Then, the ∆SST data plus the mean current temperatures (i.e. ∆SST + mean HadISST) for the region, estimated from the Hadley Centre Sea Ice and Sea Surface Temperature data set-HadISST (<https://www.metoffice.gov.uk/hadobs/hadisst/>), was integrated with the mean tolerance temperature (MTT) for marine invertebrates and fish species/functional groups (Table S2) of the food web to transfer temperature change, using the normal distribution, to the time-series of forcing function. This forcing function was then implemented in the EwE model.

**Impacts of oxygen deoxygenation**

Increasing ocean warming and stratification of the upper water column are expected to decrease the dissolved oxygen concentration in the global ocean by 1-7% during this century ^13,18-20^. Particularly, in the North Pacific Ocean, dissolved oxygen has already decreased by 22 % during the past 50 years at depths of 100 and 400 m ^19,21,22^. Deoxygenation is anomalously high in Northeastern Pacific, including declining oxygen concentrations on the British Columbia continental shelf, because of the diminishing aeriation of subsurface waters as a result of increased stratification ^14, 23, 24^. Along Canada’s Pacific coast marine ecosystems, the loss rates of oxygen decline are in the order of 0.5 to 1.0 µM/year, mainly below the surface mixed layer ^22, 23^. The oxygen decline in marine waters between 250 and 400 m depth along the British Columbia’s continental shelf affects commercial fish populations by decreasing or compressing available fish habitat with a broad spectrum of biological responses to changes in dissolved oxygen ^14^.

To calculate the forcing function for the impact of oxygen deoxygenation, the predicted times series data of dissolved oxygen was extracted from the ESM2M-GFDL for RCPs 2.6 and 8.5. Then, a dissolved oxygen index was calculated to yield changes in dissolved oxygen over time (∆DO), which was directly used as the forcing function (Figs. S4B and S5B) in the EwE model.

**Impacts of acidification (pH)**

The waters of the Northeastern Pacific Ocean have some of the lowest pH in the world because this region is at the end of the ocean’s global conveyer belt and impacted by accumulated carbon dioxide resulting from biotic respiration, built up in these ancient upwelling waters ^25, 26^. Acidification is also abnormally high in the region due to the weakening ventilation of subsurface waters resulting from increased stratification, and lack of interaction with the atmosphere ^14, 25^. Similar to the depletion of dissolved oxygen, a high range spectrum of evolutionary and biological responses to pH changes are expected ^14^. An index for changes in pH (∆pH) was calculated and used as forcing function (Figs. S4C and S5C).

**Impacts of primary production changes**

Ocean warming conditions in the ocean are expected to change productivity patterns and reduce primary production, while cold conditions will increase it in Canada’s Pacific marine ecosystems^14,27^. In 2015, for instance, a strong evidence of the impact of increased SST on productivity was observed offshore in the northeast Pacific in the form of nitrate depletion and reduced chlorophyll concentrations ^27^. Also, phytoplankton assemblages will shift toward warmer water forms ^28,29^. Changes in primary productivity were implemented based on productivity data trend from the ESM2M-GFDL for both RCP 2.6 and RCP 8.5 scenarios. A primary production index was calculated and the predicted change in phytoplankton biomass (Figs. S4D and S5D) was re-created in the EwE model by directly adding a long-term forcing function on phytoplankton that was proportional in each year to the predicted primary production trend from the ESM2M-GFD model.


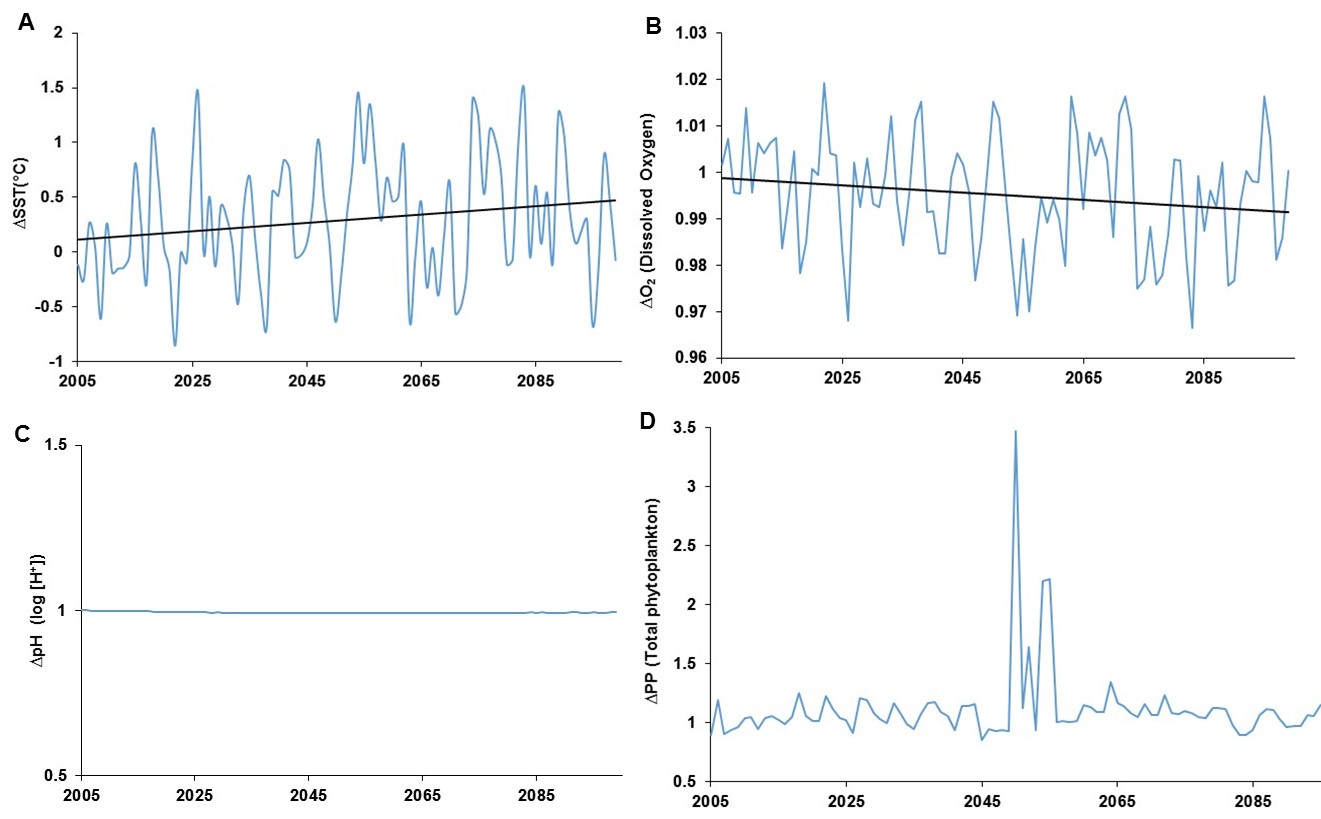


**Figure S4**. Climate change factors implemented as a forcing functions in the EwE model to include the effect of sea surface temperature (SST) changes, ocean deoxygenation (changes in dissolved oxygen, DO), ocean acidification (pH) and changes in primary productivity (PP), according to the NOAA ESM2M climate model and RCP 2.6 (optimistic scenario). (A) ∆SST is the trend of sea surface temperature difference index (i.e. SST anomalies) based on the RCP 2.6 projections; (B) ∆O_2_ is the dissolved oxygen index; (C) ∆pH is the changes in pH index; and, (D) ∆PP is the index representing the change in primary production.


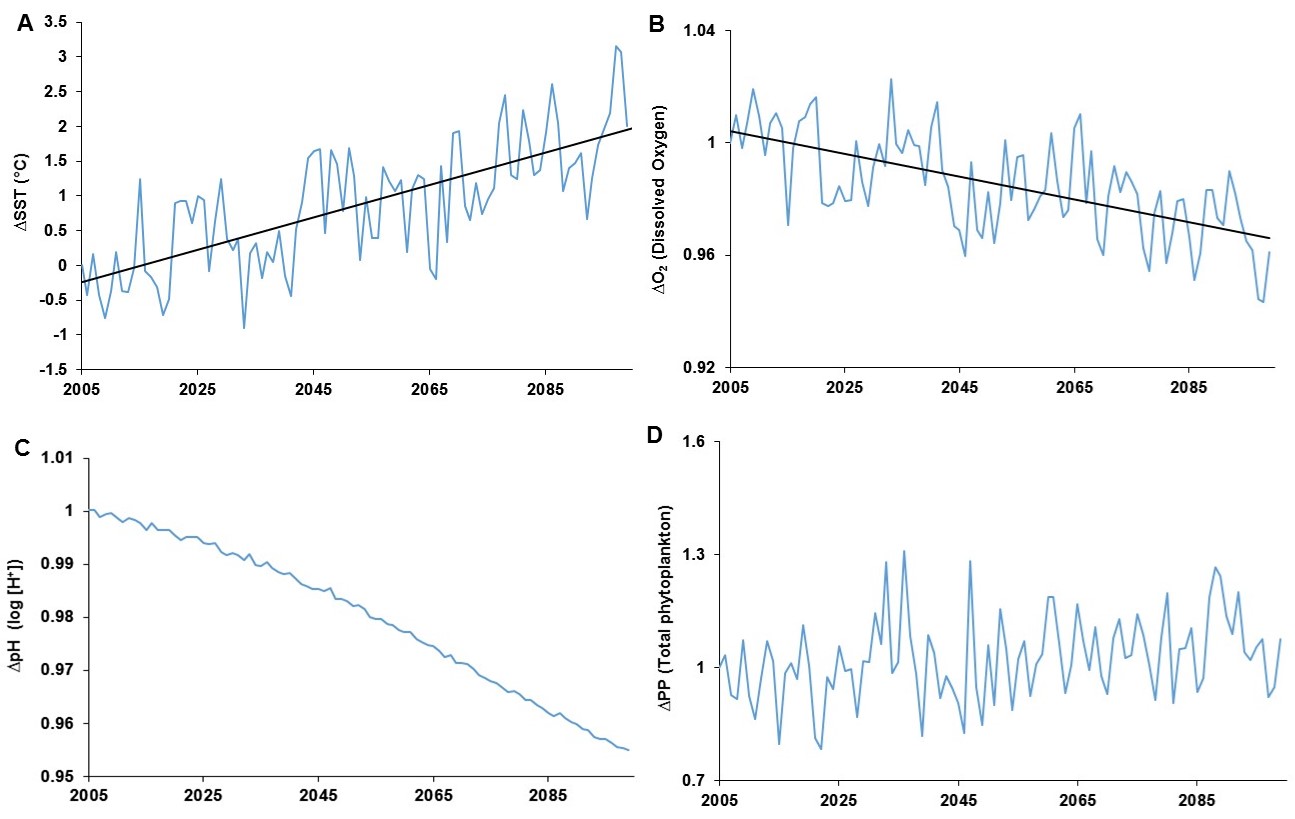


**Figure S5**. Climate change factors implemented as a forcing functions in the EwE model to include the effect of sea surface temperature (SST) changes, ocean deoxygenation (changes in dissolved oxygen, DO), ocean acidification (pH) and changes in primary productivity (PP), according to the NOAA ESM2M climate model and RCP 8.5 (High CO_2_ emissions, pessimistic scenario). (A) ∆SST is the trend of sea surface temperature difference index (i.e. SST anomalies) based on the RCP 8.5 projections; (B) ∆O_2_ is the dissolved oxygen index; (C) ∆pH is the changes in pH index; and, (D) ∆PP is the index representing the change in primary production.

**Table S2**. Mean temperature (thermal) tolerance (MTT) in °C and standard deviation (SD) for marine fish species and invertebrates included in the food web of the EwE model.

| Functional group/species | Latin name | Common name | mean Temperature (°C) | SD Temperature (°C) |
| --- | --- | --- | --- | --- |
| Sharks | *Cetorhinus maximus* | Basking shark | 15.5 | 7.13 |
|  | *Carcharodon carcharias* | Great white shark | 22.1 | 5.61 |
|  | *Prionace glauca* | Blue shark | 20.7 | 7.10 |
|  | *Alopias superciliosus* | Bigeye thresher | 25.6 | 3.01 |
|  | *Alopias vulpinus* | Thintail thresher | 22.9 | 5.92 |
|  | *Somniosus pacificus* | Pacific sleeper shark | 14.5 | 7.61 |
|  | *Galeorhinus galeus* | Tope shark | 17.8 | 3.17 |
| Pacific salmon | *Oncorhynchus gorbuscha* | Pink salmon | 5.36 | 4.11 |
|  | *Oncorhynchus keta* | Chum salmon | 5.57 | 3.32 |
|  | *Oncorhynchus nerka* | Sockeye salmon | 4.79 | 2.44 |
|  | *Oncorhynchus tshawytscha* | Chinook salmon | 6.04 | 3.61 |
|  | *Oncorhynchus kisutch* | Coho salmon | 4.77 | 2.46 |
| Pollock | *Theragra chalcogramma* | Alaska pollock | 6.17 | 3.18 |
| Hake | *Merluccius productus* | North Pacific hake | 15.9 | 3.68 |
| Rockfish | *Sebastes entomelas* | Widow rockfish | 8.52 | 3.70 |
|  | *Sebastes flavidus* | Yellowtail rockfish | 11.1 | 6.77 |
|  | *Sebastes alutus* | Pacific ocean perch | 7.01 | 3.47 |
|  | *Sebastes goodei* | Chilipepper | 15.0 | 6.32 |
|  | *Sebastes melanops* | Black rockfish | 14.9 | 3.61 |
|  | *Sebastes paucispinis* | Bocaccio | 7.52 | 3.98 |
|  | *Sebastes pinniger* | Canary rockfish | 7.54 | 3.83 |
|  | *Sebastolobus alascanus* | Shortspine thornyhead | 7.24 | 3.98 |
|  | *Scorpaenichthys marmoratus* | Cabezon | 12.9 | 6.93 |
| Flatfish | *Hippoglossus stenolepis* | Pacific halibut | 6.60 | 3.33 |
|  | *Atheresthes stomias* | Arrowtooth flounder | 8.40 | 6.21 |
|  | *Citharichthys sordidus* | Pacific sanddab | 13.8 | 15.9 |
|  | *Eopsetta jordani* | Petrale sole | 8.43 | 3.14 |
|  | *Glyptocephalus zachirus* | Rex sole | 7.14 | 3.77 |
|  | *Microstomus pacificus* | Dover sole | 10.6 | 7.36 |
|  | *Parophrys vetula* | English sole | 9.60 | 3.14 |
|  | *Platichthys stellatus* | Starry flounder | 4.55 | 4.22 |
|  | *Psettichthys melanostictus* | West American sand sole | 9.04 | 3.24 |
|  | *Lepidopsetta bilineata* | Rock sole | 6.30 | 3.42 |
| Foragefish | *Sardinops sagax* | South American pilchard | 20.2 | 6.16 |
|  | *Clupea pallasii* | Pacific herring | 7.43 | 4.52 |
|  | *Alosa sapidissima* | American shad | 11.4 | 4.46 |
|  | *Engraulis mordax* | Californian anchovy | 16.2 | 4.66 |
| Squid | *Loligo opalescens* | California market squid | 15.2 | 3.27 |
| Zoobenthos | *Crangon crangon* | Common shrimp | 11.6 | 3.49 |
|  | *Cancer magister* | Dungeness crab | 10.1 | 4.33 |
|  | *Pandalus borealis* | Northern prawn | 5.41 | 4.37 |

**Contaminant Data: PCBs and mercury (MeHg)**

Input contaminant data was retrieved from the best data available for PCBs and MeHg (Fig. S3). The data for PCB concentrations used in the Ecotracer module were obtained from different peer reviewed publications, and represent the baseline data collected from the mid-1990s to the late 2000s. The initial PCB load per area (g/m^2^), base inflow rate (g/m^2^/day) and volume exchange loss (1/day) for PCBs in the marine environment were estimated from data reported for surface sediments and 10 years inventories of total PCBs in the Strait of Georgia ^30^. Similarly, using sedimentation rates (0.3 to 3 cm/yr) and depths of sediment surface mixed layers (4 cm to 25 cm) determined for 18 sites in the Strait of Georgia ^31,32^, the half-life (*t* _1/2_) of PCB in the surface mixed layer, before it becomes permanently buried, can be calculated, i.e. 10 ± 8 years (mean ± SD) or 3650 ± 2920 days ^31,32^. Based on this half-life (i.e. *t ½* = ln 2/ *k_E_*), the approximate elimination or decay rate (*k_E_*) is 0.0693/yr or 0.00019/day.

Data for PCBs was available for some functional groups or marine species of the food web, including zooplankton, Chinook salmon, harbor seal, Steller sea lions and both resident and transient killer whales ^33-40^, as reported in Table S3. Sediment concentrations for PCBs measured in the Strait of Georgia^41^ were also used as data input and assumed to be representative for detritus. For sediments, PCBs refer to the sum of a full suite of 209 PCB congeners (∑PCB) reported in Johannessen *et al*.^32^. For killer whales, total PCB concentrations was calculated as the ∑PCB of 136 congeners as reported in Ross *et al*.,^33^ most of which are recalcitrant congeners with high K_OW_, including non-ortho PCBs (i.e. 77, 81, 126, 169) and mono-ortho PCBs (i.e. 105, 114, 118, 123, 156, 157, 167, 189), among other congeners. For Chinook salmon, ∑PCB include the sum of the concentration of 135 PCB congeners as reported in Cullon *et al.* ^36^. Data on biological uptake and metabolic rates for PCBs were retrieved from Alava *et al*.^3^ and also entered in Ecotracer (Table S3).

In contrast to PCBs, empirical data sets for mercury, mainly for MeHg, were limited for the study region. The initial mercury load per area (g/m^2^), base inflow rate (g/m^2^/day) and volume exchange loss (1/day) in the marine environment were estimated from Johannessen *et al.*^31^. As aforementioned for PCBs, sedimentation rates and depths of sediment surface mixed layers were estimated from historical trends in mercury sedimentation and mixing in the Strait of Georgia, Canada ^31^ and used to calculated the half-life (*t* _1/2_) of Hg in the surface mixed layer (i.e. ≈10 years).

Organic mercury (MeHg) data for biota was available for wild Chinook, chum and coho salmon species collected and sampled across a range of sampling dates and geographical locations from coastal in BC in 2003 ^42^, as well as MeHg data measured in hair of adult harbor seals captured at 10 sites in BC (Canada) and Washington State (USA) between 2003 and 2010 ^43^. For harbor seals, we assumed that 90% of total mercury (THg) measured was MeHg. Surface sediment mercury concentrations measured from seventeen sediment cores collected in the Strait of Georgia ^31^ were used as data input and assumed to be representative for detritus. Mercury concentrations in surface sediments from the coastal marine environment of the Strait of Georgia were assumed to be representative of “detritus” for the purpose of the modelling work as surficial sediments are biologically active and represent current concentrations, serving as a sink or/and source of exposure to the food web in response to changes of mercury inputs and cycling in the coastal-marine environment the Strait of Georgia^31^. We considered and worked under this assumption as the modelling work was focused on a coastal-marine area and not in the deep ocean, where detritus concentrations of mercury can indeed be higher than coastal sediment concentration. Data on biological uptake and metabolic rates (demethylation rates) were retrieved from Booth and Zeller ^44^ or adapted from Alava *et al*.^3^ (Table S4). For simulations, average concentrations for PCBs and mercury (i.e. MeHg) reported in these studies was entered as initial concentrations for biota and detritus in the Ecotracer input module (Tables S3 and S4) coupled with climate change forcing functions above described.

**Table S3**. Empirical PCB concentration data and units entered as input in the Ecotracer module. PCB concentration data for marine biota were retrieved from different sources reported elsewhere^33-40^. Uptake and metabolic biotransformation rates and contamination assimilation were based on Alava *et al*.^3^ and Hickie *et al*.^53^.

| Functional group name/species | Initial concentration (mg/kg lipid weight) | Concentration in immigrating biomass (mg/kg) | Direct (dietary) uptake rate (g/g/day) | Metabolic biotransformation rate or k_M_ (per day) | Proportion of contaminant assimilated [0-1] |
| --- | --- | --- | --- | --- | --- |
| Transient killer whales* | 219.0 | 0 | 0 | 0.000055 | 1.00 |
| Southern resident killer whales* | 78.2 | 0 | 0.049 | 0.000055 | 1.00 |
| Baleen Whales | 0 | 0 | 0 | 0.00 | 1.00 |
| Small Cetaceans | 0 | 0 | 0 | 0.00 | 1.00 |
| Steller sea lions | 2.30 | 0 | 0 | 0.00 | 1.00 |
| Harbour seals | 1.29 | 0 | 0 | 0.00 | 1.00 |
| Sharks | 0 | 0 | 0 | 0.00 | 1.00 |
| Chinook Salmon | 1.13 | 0 | 0.0110 | 0.00 | 1.00 |
| Chum Salmon | 0 | 0 | 0.012 | 0.00 | 1.00 |
| Coho Salmon | 0 | 0 | 0.011 | 0.00 | 1.00 |
| Pink Salmon | 0 | 0 | 0 | 0.00 | 1.00 |
| Hake | 0 | 0 | 0 | 0.00 | 1.00 |
| Pollock | 0 | 0 | 0.019 | 0.00 | 1.00 |
| Rockfish | 0 | 0 | 0 | 0.00 | 1.00 |
| Flatfish | 0 | 0 | 0.020 | 0.00 | 1.00 |
| Squids | 0 | 0 | 0.022 | 0.00 | 1.00 |
| Forage Fishes | 0 | 0 | 0.024 | 0.00 | 1.00 |
| Zooplankton | 0.14 | 0 | 0.061 | 0.00 | 1.00 |
| Zoobenthos | 0 | 0 | 0.05 | 0.00 | 1.00 |
| Phytoplankton | 0 | 0 | 0.547 | 0.00 | 1.00 |
| Detritus (i.e. surficial sediment)****** | 0.26 | 0 | 0 | 0.00 | 1.00 |

*****Initial concentrations for killer whales were changed to 3.0 mg/kg lipid through iterations because of systematic overprediction yielded by the simulation and to be comparable to observed PCB concentrations in killer whale. See model bias (MB) section below.

******PCB concentration in detritus were normalized to the Total Organic Carbon (TOC = 4.3%) content in surficial sediments

**Table S4**. Empirical MeHg concentration data and units entered as input in the Ecotracer module. Mercury concentration data for marine biota were retrieved from different sources reported elsewhere^42, 43^. Dietary/uptake rates were based on Alava *et al.*^3^, while metabolic biotransformation/demethylation rates were based on Booth and Zeller^44^. MeHg assimilation was based on the review by Bradely *et al*.^61^.

| Functional group name/species | Initial concentration (mg/kg wet weigth) | Concentration in immigrating biomass (mg/kg) | Direct (dietary) uptake rate (g/g/day) | Metabolic biotransformation rate or k_M_ (per day) | Proportion of contaminant assimilated [0-1] |
| --- | --- | --- | --- | --- | --- |
| Transient killer whales | 0 | 0 | 0.049 | 0.0003 | 1.00 |
| Southern resident killer whales | 0 | 0 | 0.049 | 0.0003 | 1.00 |
| Baleen Whales | 0 | 0 | 0 | 0.000055 | 1.00 |
| Small Cetaceans | 0 | 0 | 0 | 0.000055 | 1.00 |
| Steller sea lions | 0 | 0 | 0 | 0.001 | 1.00 |
| Harbour seals***** | 7.5 | 0 | 0 | 0.001 | 1.00 |
| Sharks | 0 | 0 | 0 | 0.00 | 1.00 |
| Chinook Salmon | 0.1 | 0 | 0.0110 | 0.00 | 1.00 |
| Chum Salmon | 0.018 | 0 | 0.012 | 0.00 | 1.00 |
| Coho Salmon | 0.059 | 0 | 0.011 | 0.00 | 1.00 |
| Pink Salmon | 0.012 | 0 | 0 | 0.00 | 1.00 |
| Hake | 0 | 0 | 0 | 0.00 | 1.00 |
| Pollock | 0 | 0 | 0.019 | 0.00 | 1.00 |
| Rockfish | 0 | 0 | 0 | 0.00 | 1.00 |
| Flatfish | 0 | 0 | 0.020 | 0.00 | 1.00 |
| Squids | 0 | 0 | 0.022 | 0.00 | 1.00 |
| Forage Fishes | 0 | 0 | 0.024 | 0.00 | 1.00 |
| Zooplankton | 0 | 0 | 0.00001 | 0.00 | 1.00 |
| Zoobenthos | 0 | 0 | 0.000025 | 0.00 | 1.00 |
| Phytoplankton | 0 | 0 | 0.00001 | 0.00 | 1.00 |
| Detritus (i.e. surficial sediment)****** | 3.155 | 0 | 0 | 0.00 | 1.00 |

*****Mercury concentration data from hair of adult harbor seals was originally reported in dry weight (dw) basis. This concentrations value was assumed for modelling purposes because concentrations in a wet weight basis and the wet fractions were not reported in the publication (Nöel et al., 2016).

******MeHg concentration in detritus were normalized to the Total Organic Carbon (TOC = 4.3%) content in surficial sediment

**Ecopath with Ecosism (EwE) and Ecotracer model**

As shown in Figure S3, The modeling approach relies on the application and simulations of the Ecopath with Ecosism (EwE) ecosystem model with the indirect inclusion of four climate change forcing factors (i.e. ocean temperature, acidification, dissolved oxygen and primary production) that are predicted to change with climate change ^10,11,13^ and Ecotracer, which is the modelling module to track and assess the bioaccumulation of pollutants in marine food webs over time ^45^.

EwE is a trophodynamic simulation modelling integrating biotic and abiotic components of the ecosystem, based on assumptions of mass balance and a system of linear equations describing and tracking the average flows of mass and energy between functional groups (i.e. biomass pools: species or groups of species aggregated according to life-history and niche characteristics) according to a diet composition matrix, while accounting for energy lost in respiration, emigration, and decomposition through time ^45-47^. Details on the core principles and equations of EwE can be found in the EwE user guide available online ^48^.

Ecotracer uses Ecosim to predict movement and accumulation of contaminants, including persistent organic pollutants (POPs) and mercury in food webs ^44,49,50^. Specifically, changes in concentrations of chemicals are predicted using flow rates from Ecosim along with decay or elimination rates and physical exchange rates ^48^. The linear dynamical equation for time changes in contaminant concentration in a given functional group (pool) or species *i* is expressed as:

C*iBi*/*dt* = (*Cj •* GC*i • Qji / Bj*) + (*ui* • *Bi • Co*) + (*ci • Ii*) – [(C*i* • Q*ij* / B*i*) + C*i* • *MOi* + ((1-

GC*i*) • *∑jCj • Qji/Bj + ei • Ci + di • Ci*]

Thus, the time dynamic changes in contaminant concentration in the biomass of a given functional group or species *i* (*CiBi*) can explicitly be described by the following components, based on Christensen and Walters ^45^:

1. Uptake from food: Cj • GCi • Qji / Bj where Cj = conc in food j, GCi = proportion of food assimilated by type i organisms; Qji = biomass flow rate from j to i (estimated in Ecopath as Bi • (Q/B)I • DCij) i, Bj=food j biomass;
2. Direct uptake from environment: ui • Bi • Co, where ui=parameter representing uptake per biomass per time, per unit environmental concentration, Bi=biomass, Co=environmental concentration;
3. Concentration in immigrating organisms: ci • Ii, where ci = parameter (tracer per unit biomass in immigrating biomass), Ii = biomass of pool i immigrants per time;
4. Predation: Ci • Qij / Bi, where Ci=concentration in pool i, Qij = consumption rate of type i organisms by predator type j, Bi = biomass in pool i;
5. Detritus: Ci • MOi + (1-GCi) • ∑*j*C*j* • Qji / Bj, where MOi = non-predation death rate of type i (per year), GCi = fraction of food intake assimilated, Qji = intake rate if type j biomass by type i;
6. Emigration: ei • Ci, where ei = emigration rate (per year);
7. Metabolism: di • Ci, where di = metabolism + decay rate for the material while in pool i.

For the purpose of the modeling work, the contaminant concentration in immigrating organisms (*ci•Ii*) and emigration (*ei• Ci*) were considered to be negligible (i.e. set to zero) for the regional food web of the southern resident killer whales. In doing so, the equation is simplified as:

*CiBi*/*dt* = (*Cj •* GC*i • Qji / Bj*) + (u*i* • *Bi • Co*) – [(C*i* • Q*ij* / B*i*) + C*i* • *MOi* + ((1-

GC*i*) • *∑jCj • Qji/Bj + di • Ci*]

**Model Bias Performance Evaluation**

To examine the performance of the modelling approach, a model bias (*MB*) approach was implemented in our modelling approach (Figure S3). The simulation model (Ecotracer subroutine) was preset for earlier time steps (1930-2010) and run forward to perform and predict steady-state historical concentrations of PCBs, which was used here as our standard reference contaminant, to fit available contaminant data (i.e. PCBs) observed in males of SRKW, from 1993 to 1996 ^33^ and from 2004 to 2006 ^35^, as well as in Chinook salmon, from August and October 2000 to June and September 2001 in the Strait of Georgia, BC, Canada ^36^, and from 1992 to1996 in Puget Sound, WA, USA ^51^. SRKW females were excluded due to the cofounding nature and influence of age-sex class and reproductive factors such as pregnancy and lactation in reproductive females (i.e. maternal transfer) on contaminant accumulation and mobilization ^33,35, 52^. Initial PCB concentrations in biota biomass were preset at zero as pollutant concentration per biomass are not required to explore long-term equilibrium concentrations and steady state, while allowing the biological functional groups to equilibrate ^44, 50^. An initial sediment (detritus) concentration of 0.30 mg/kg (i.e. approximate initial spike of PCBs in sediments in 1930, based on the ratio of estimated PCB sediment core data for the Georgia Basin of the coastal marine region of BC, reported by Hickie *et al*. ^53^; see Figure S3 in supporting information in ref. 53) was entered to initiate the simulation. This simulation allowed the historical projection of PCB data for industrialized period estimates in biota when PCB was still used (1930-1970), preceding the phasing out of this contaminant in North America in the late 1970s. To be comparable to the empirical data reported in a lipid weight basis, the historical projections of PCB data were lipid normalized using a set of values of lipid content for SRKW (i.e. 9.6%, 24%, 40%) and Chinook salmon (i.e. 0.87%, 4.0%, 6.4%, 10%), as documented elsewhere ^33-35, 51^.

Model performance was quantitatively assessed for total PCBs, i.e. the sum of PCB congener concentrations (ΣPCBs), in the form of the mean *MB*, which is derived for each species as^3^:

$$MB_{i}=10^{\left(\sum_{i=1}^{n} \frac{\left[\log(C_{BP,i\Sigma PCB}\text{/}C_{BO,i\Sigma PCB}\text{)}\right]}{n}\right)}$$

where *C*_BP,_*_i_*_ΣPCB_ and *C*_BO,_*_i_*_ΣPCB_ are, respectively, the model calculated and observed ∑PCB concentrations in each species *i* for observations *n* ranging from *n* = 1 to the total number of concentration measurements. Assuming a log-normal distribution of the ratio *C*_BP,_*_i_*_ΣPCB_ / *C*_BO,_*_i_*_ΣPCB_, *MB*_j_ is the geometric mean of the ratio of predicted and observed concentrations (*C*) for ΣPCB in species *i*. *MB* indicates the model’s systematic over- (*MB*>1) or under-prediction (*MB*<1) of the concentrations (*C*) for ΣPCB. The variability of over- and under-estimation of measured values is represented by the standard deviation (±SD) of *MB* (*MB*_SD_), and is an indication of the variability and uncertainty of model predictions. The error of *MB* can be described as a factor (rather than a term) of the geometric mean because of the log-normal distribution of the ratio of predicted and observed ΣPCB.

**Apparent Trophic Magnification Factor**

The trophic magnification factor (TMF), a bioaccumulation metric that is often used to express the biomagnification of pollutants in an entire food-web ^54-56^ was determined for the simulations (i.e. 2005-2100). The TMF was calculated as the antilog of the regression slope (i.e. TMF =10*^b^*, where *b* is the slope) of the linear regression between the log transformed concentrations of the contaminant (i.e. PCBs, MeHg) predicted in organisms of the food web and their trophic level (TL) (i.e. log [Hg] = *a* + *b*TL; or log [PCBs] = *a* + *b*TL) ^54^.

The TMF (slope, *b*) was statistically evaluated using a significance level (α) of 0.05. A TMF > 1 (*b >* 0) indicates that the contaminant biomagnifies in the food-web. A TMF < 1 (*b < 0*) indicates trophic dilution of the contaminants, while a TMF=1 (*b* =0) indicates no change in contaminant concentrations among organisms of a food web ^54^.

While the major results of this work are mainly focused on the projections found in the top predator of the food web (i.e. southern resident killer whale; hereafter referred to as SRKW) and its major prey (i.e. Chinook salmon), we provided evidence of bioaccumulation for other organisms, and examined pollutant biomagnification in the food web by calculating the apparent TMF, for the simulation time frame within both RCP scenarios. Although the application of the TMF is becoming a new practical approach for simulations involving food web bioaccumulation modeling^8^, the trophic magnification patterns predicted here for PCBs and MeHg serve as an additional index to investigate the influence climate change on pollutant bioaccumulation, echoing the magnitude of its impact in food web biomagnification.

The highest TMFs are generally found for organic contaminants that are slowly metabolized by animals, i.e. metabolic rate (*k_M_*) < 0.01/day ^57^. TMFs are more variable in marine food webs relative to those from freshwaters with the highest values found in long food webs, including endotherms and containing multiple predators ^54,56,57^.

**Implications for public health**

Our simulations showed that projected MeHg concentrations in forage fish (e.g., herring, sandlance), squid and Chinook salmon super pass the Canada’s maximum level (ML) for mercury consumption limits of 0.5-1.0 mg/kg wet weight (Figs. 2C-2E), as well as exceeded the consumption advisory level of 0.5 mg/kg recommended by the World Health Organization ^58^. Exacerbation of MeHg bioaccumulation was predicted under the impact of climate change scenarios in fish species and pilot whales from the Northeast Atlantic with an associated increase of human exposure to mercury from the consumption of whales ^44^.This has obvious implications for food security and safety due to potential contamination and toxic effects ^59^, mainly for coastal aboriginal people and First Nations’ communities consuming high amount of fish (64% of consumed seafood is salmon) and seafoods, i.e. 15 times the average Canadian consumers ^60^.


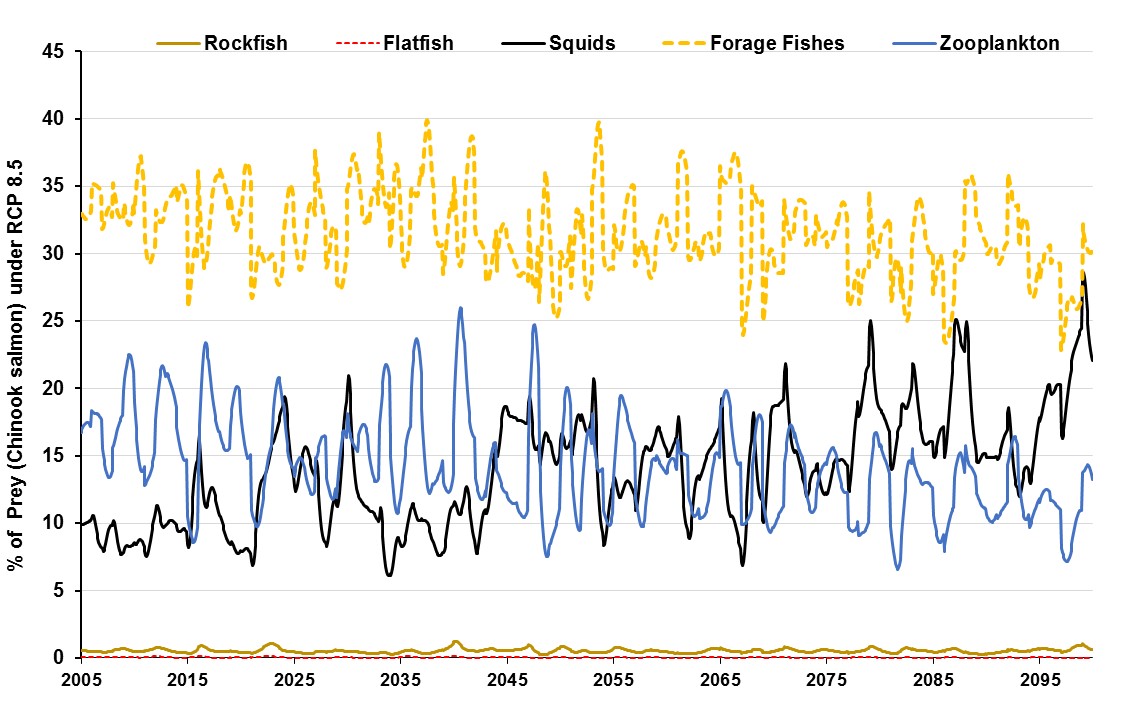


**Figure S6**. Simulation illustrating the trends of the percentage of prey of Chinook salmon influenced by climate change forcing under the business as usual/ high emissions scenario (i.e. RCP 8.5 scenario) from 2005 to 2100. Under this scenario, the biomass of squid consistently increased in comparison to a relative decrease in biomass for both foragefish and zooplankton.


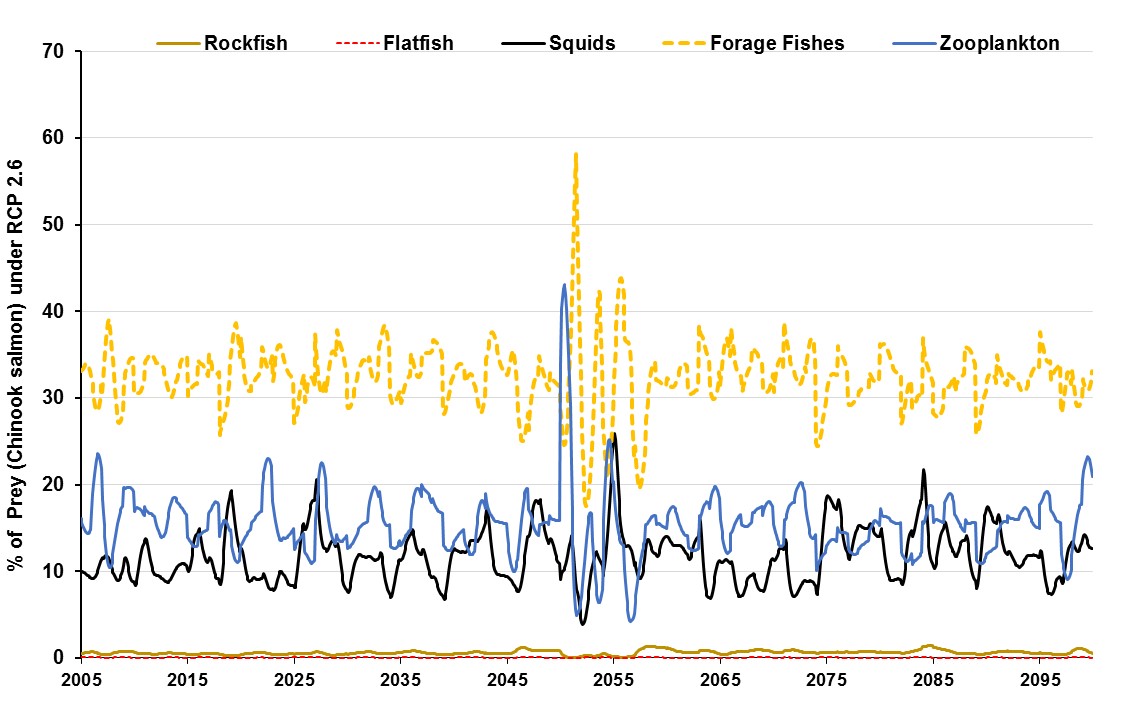


**Figure S7**. Simulation illustrating the trends of the percentage of prey of Chinook salmon influenced by climate change forcing under the business as usual/ high emissions scenario (i.e. RCP 2.6 scenario) from 2005 to 2100. Under this scenario, the biomass of squid, foragefish and zooplankton is relatively constant, except for some inter-temporal pulses between 2050 and 2060.


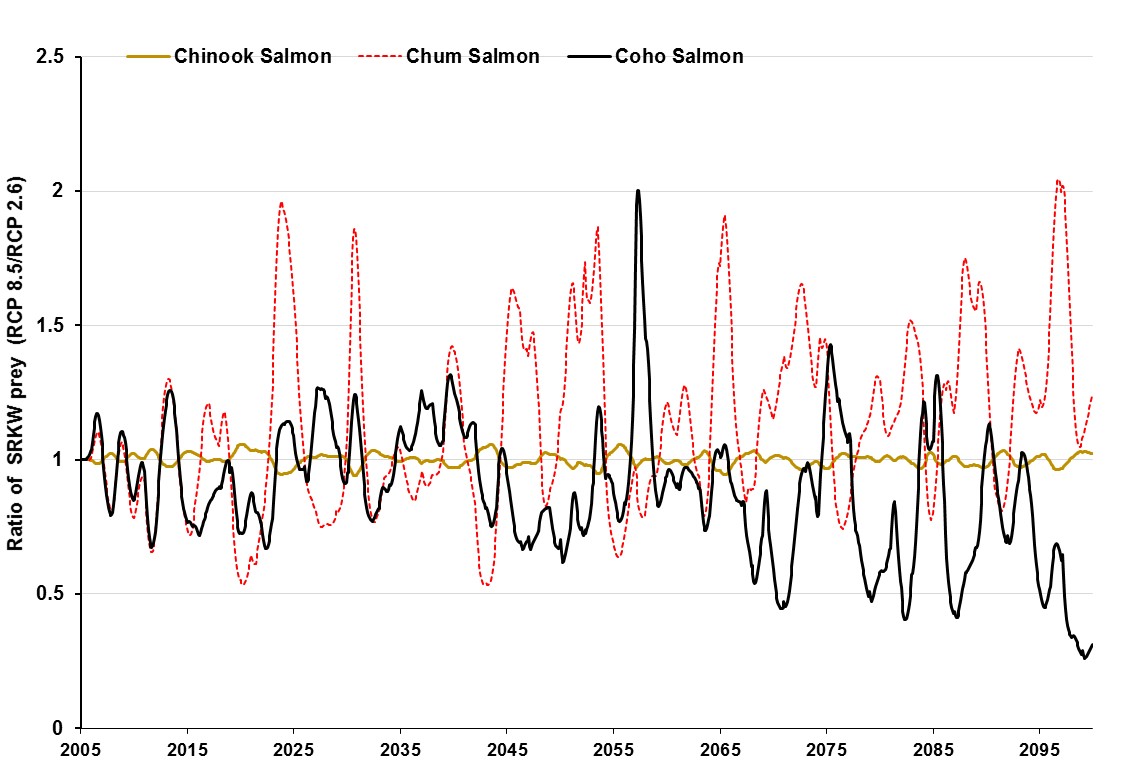


**Figure S8**. Simulation illustrating the trends of the ratio of prey proportion (i.e. RCP 8.5/RCP 2.6 ratio) for southern resident killer whales (SRKW) projected with the EwE model. The brown solid line is the projected ratio of Chinook salmon (SRKW’s major diet items) over the simulation period. The black solid line is the projected declining prey ratio trend for Coho salmon, while the red dotted line is the projection ratio with relatively positive trend for chum salmon at the end of the century.


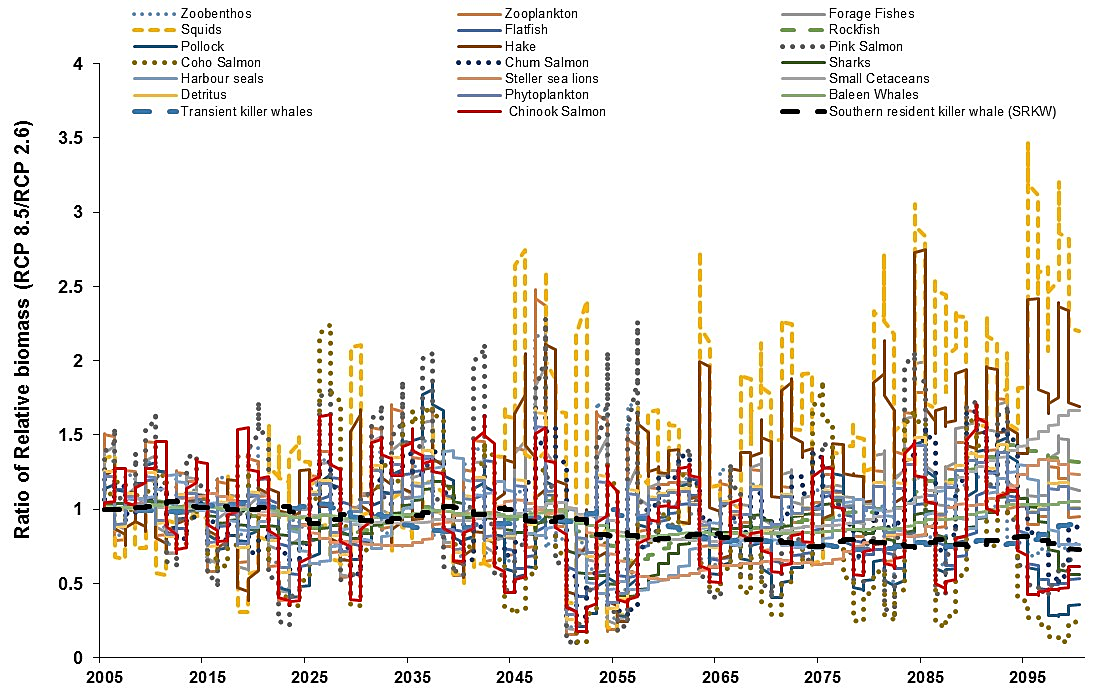


**Figure S9**. Simulation illustrating the trends of the ratio of relative biomass (i.e. RCP 8.5_Biomass_/RCP 2.6_Biomass_) projected with the EwE model for species/functional groups of the marine food web. The black dashed line is the projected declining biomass ratio trend for southern resident killer whales (SRKW), while the red solid line is the projected declining biomass ratio for Chinook salmon (SRKW’s major diet items) at the end of the century. The biomass ratio of other Pacific salmon species (i.e., Coho, chum and pink salmon), encompassing a small proportion of the diet for SRKW, is also projected to decline. For instance, the brown, blue and grey dotted lines represent the biomass trends for Coho, chum and pink salmon, respectively. Contrasting to these trends, the squid biomass ratio is projected to increase (see yellow dashed line).


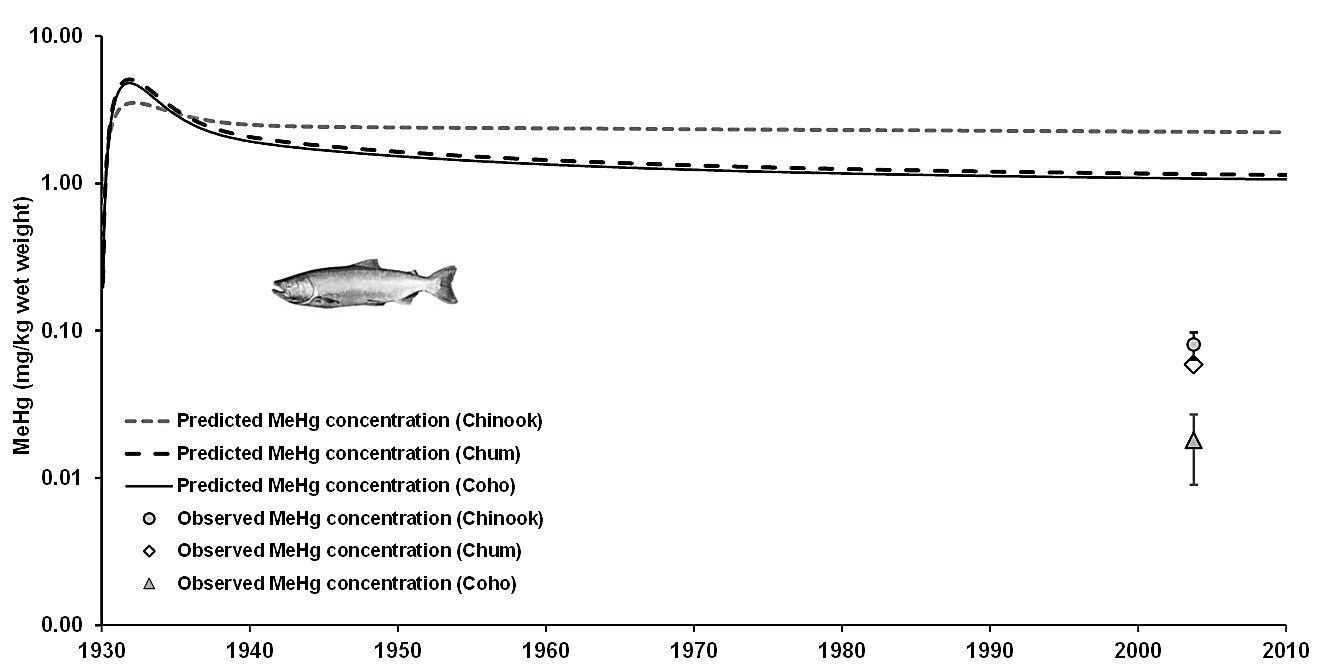


**Figure S10**. Historical projections of MeHg concentrations (mg/kg wet weight) in Chinook, chum and coho salmon simulated with the Ecotracer routine of the EwE model. For comparison purposes, observed MeHg concentrations for Chinook, chum and coho salmon from the Strait of Georgia reported by Kelly et al. (2008) are also plotted. The closed circle, open jack and grey triangle are the average PCB concentration observed in Chinook, chum and coho salmon. Error bars are 95% CI.

**Table S5**. Calculation of the model bias (MB) to test the performance of the model using predicted (*C*_BP,_*_i_*_ΣPCB_) and observed (*C*_BO,_*_i_*_ΣPCB_) data for PCBs in southern resident killer whales. The predicted PCB data generated from the EwE model (Ecotracer) under steady state conditions and no climate change forcing were normalized to observed values of the lipid content (i.e. 9.6%, 24% and 40%) measured in southern resident killer whales to be comparable with empirical, lipid normalized PCB data.

|  | Lipid (24%) | | | Lipid (9.6%) | | | Lipid (40%) | | |
| --- | --- | --- | --- | --- | --- | --- | --- | --- | --- |
|  | log *C*_BP,_*_i_*_ΣPCB_ | log *C*_BO,_*_i_*_ΣPCB*_ | log *C*_BP,_*_i_*_ΣPCB_ - log *C*_BO,_*_i_*_ΣPCB_ | log *C*_BP,_*_i_*_ΣPCB_ | log *C*_BO,_*_i_*_ΣPCB*_ | log *C*_BP,_*_i_*_ΣPCB_ - log *C*_BO,_*_i_*_ΣPCB_ | log *C*_BP,_*_i_*_ΣPCB_ | log *C*_BO,_*_i_*_ΣPCB*_ | log *C*_BP,_*_i_*_ΣPCB_ - log *C*_BO,_*_i_*_ΣPCB_ |
| 1993-1996  (Ross et al., 2000) | 1.72 | 2.17 | -0.44 | 2.12 | 2.17 | -0.04 | 1.50 | 2.17 | -0.66 |
| 2004 & 2006 (Krahn et al. 2007) | 2.04 | 1.82 | 0.22 | 2.44 | 1.82 | 0.62 | 1.82 | 1.82 | 0.00 |
| Mean |  |  | -0.11 |  |  | 0.29 |  |  | -0.33 |
| SD |  |  | 0.47 |  |  | 0.47 |  |  | 0.47 |
| MB(geomean) |  |  | 0.8 |  |  | 1.9 |  |  | 0.5 |
| MB_SD_ |  |  | 2.9 |  |  | 2.9 |  |  | 2.9 |

*Observed PCB data (mg/kg lipid weight) in southern resident killer whales were retrieved from Ross et al. (2000) and Krahn et al. (2007).

**Table S6**. Calculation of the model bias (MB) to test the performance of the model using predicted (*C*_BP,_*_i_*_ΣPCB_) and observed (*C*_BO,_*_i_*_ΣPCB_) data for PCBs in Chinook salmon. The predicted PCB data generated from the EwE model (Ecotracer) under steady state conditions and no climate change forcing were normalized to observed values of the lipid content (i.e. 0.87%, 4% , 6.38% and 10%) measured in Chinook salmon to be comparable with empirical, lipid normalized PCB data.

|  | Lipid (0.87%) | | | Lipid (4.0%) | |  | Lipid (6.38%) | | | Lipid (10%) | | |
| --- | --- | --- | --- | --- | --- | --- | --- | --- | --- | --- | --- | --- |
|  | log *C*_BP,_*_i_*_ΣPCB_ | log *C*_BO,_*_i_*_ΣPCB*_ | log *C*_BP,_*_i_*_ΣPCB_ - log *C*_BO,_*_i_*_ΣPCB_ | log *C*_BP,_*_i_*_ΣPCB_ | log *C*_BO,_*_i_*_ΣPCB*_ | log *C*_BP,_*_i_*_ΣPCB_ - log *C*_BO,_*_i_*_ΣPCB_ | log *C*_BP,_*_i_*_ΣPCB_ | log *C*_BO,_*_i_*_ΣPCB*_ | log *C*_BP,_*_i_*_ΣPCB_ - log *C*_BO,_*_i_*_ΣPCB_ | log *C*_BP,_*_i_*_ΣPCB_ | log *C*_BO,_*_i_*_ΣPCB*_ | log *C*_BP,_*_i_*_ΣPCB_ - log *C*_BO,_*_i_*_ΣPCB_ |
| August & September 1992-1996 (O’Neill and West 2009) | 1.27 | 0.19 | 1.08 | 0.61 | 0.19 | 0.41 | 0.41 | 0.19 | 0.21 | 0.21 | 0.19 | 0.01 |
| August & October 2000 (Cullon et al., 2009) | 1.26 | 0.05 | 1.21 | 0.60 | 0.05 | 0.55 | 0.40 | 0.05 | 0.35 | 0.20 | 0.05 | 0.15 |
| June &September 2001 (Cullon et al., 2009) | 1.26 | -0.07 | 1.33 | 0.60 | -0.07 | 0.67 | 0.40 | -0.07 | 0.47 | 0.20 | -0.07 | 0.27 |
| Mean |  |  | 1.21 |  |  | 0.54 |  |  | 0.34 |  |  | 0.14 |
| SD |  |  | 0.13 |  |  | 0.13 |  |  | 0.13 |  |  | 0.13 |
| MB(geomean) | |  | 16.1 |  |  | 3.49 |  |  | 2.2 |  |  | 1.4 |
| MB_SD_ | |  | 1.34 |  |  | 1.34 |  |  | 1.34 |  |  | 1.34 |

*Observed PCB data (mg/kg lipid weight) in southern resident killer whales were retrieved and averaged from Cullon et al. (2009) and O’Neill and West (2009).

**Table S7**. Model bias (MB) ratio comparing projected data from simulations versus observed MeHg data in Pacific salmon species. Measured concentrations of MeHg for Chinook, chun and coho were retrieved from Kelly et al (2008).

| Mean Predicted MeHg concentrations | | | Mean Observed MeHg concentrations | | | MB ratio = Predicted [MeHg] in salmon/  Observed [MeHg] in salmon | | |
| --- | --- | --- | --- | --- | --- | --- | --- | --- |
| Chinook | Chum | Coho | Chinook | Chum | Coho | Chinook | Chum | Coho |
| 2.24 | 1.16 | 1.08 | 0.08 | 0.02 | 0.06 | 27.8 | 64.2 | 18.3 |

**Table S8**. Regression statistics for the linear regression models of the mean log-transformed contaminant data projected with simulations over the period 2005-2100 versus trophic levels (TL) in marine organisms of the food web under RCP 2.6, 8.5 and no-climate change scenarios, as well as calculations of the trophic magnification factors (TMFs) for PCBs and MeHg.

| Scenarios | Contaminant | *r^2^* | *r* | Slope (b) | | TMF* | *p*-value | Outcome |
| --- | --- | --- | --- | --- | --- | --- | --- | --- |
| No climate change | PCBs | 0.534 | 0.730 | 0.721 | 5.27 | | 0.00299** | PCB biomagnification |
| RCP2.6 | PCBs | 0.529 | 0.727 | 0.698 | 4.99 | | 0.00322** | PCB biomagnification |
| RCP8.5 | PCBs | 0.530 | 0.728 | 0.707 | 5.09 | | 0.00317** | PCB biomagnification |
| No climate change | MeHg | 0.708 | 0.841 | 1.725 | 53.1 | | 0.000161** | MeHg biomagnification |
| RCP2.6 | MeHg | 0.708 | 0.841 | 1.721 | 52.7 | | 0.000162** | MeHg biomagnification |
| RCP8.5 | MeHg | 0.703 | 0.838 | 1.720 | 52.4 | | 0.000181** | MeHg biomagnification |

* Calculated as the anti-log of the slope: TMF = 10^b^, where b is the slope.

** Double asterisks denote a high significant relationship.

**Appendix I**

Projections of the RCP 2.6 and RCP 8.5 (1950-2099) retrieved from the Earth System Model using Modular Ocean Model (ESM2M)/NOAA- GFDL (Dunne et al., 2012) for climate change factors. Data from 1996 to 2099 was used for the EwE modeling work for the studied region.

|  | RCP 2.6 | | | | RCP 8.5 | | | |
| --- | --- | --- | --- | --- | --- | --- | --- | --- |
| **Year** | SST (°C) | Dissolved  oxygen | pH [H^+^] | Total phytoplankton | SST (°C) | Dissolved  oxygen | pH [H^+^] | Total phytoplankton |
| **1950** | 10.79583 | 0.00027 | 7.070E-09 | 0.07848 | 10.79583 | 0.00027 | 7.070E-09 | 0.07848 |
| **1951** | 10.99733 | 0.00027 | 7.064E-09 | 0.07081 | 10.99733 | 0.00027 | 7.064E-09 | 0.07081 |
| **1952** | 11.08835 | 0.00027 | 7.122E-09 | 0.06812 | 11.08835 | 0.00027 | 7.122E-09 | 0.06812 |
| **1953** | 10.68208 | 0.00027 | 7.044E-09 | 0.07307 | 10.68208 | 0.00027 | 7.044E-09 | 0.07307 |
| **1954** | 10.33352 | 0.00027 | 7.048E-09 | 0.07562 | 10.33352 | 0.00027 | 7.048E-09 | 0.07562 |
| **1955** | 10.10804 | 0.00028 | 6.981E-09 | 0.09268 | 10.10804 | 0.00028 | 6.981E-09 | 0.09268 |
| **1956** | 10.07429 | 0.00028 | 7.017E-09 | 0.08644 | 10.07429 | 0.00028 | 7.017E-09 | 0.08644 |
| **1957** | 11.18333 | 0.00027 | 7.316E-09 | 0.07029 | 11.18333 | 0.00027 | 7.316E-09 | 0.07029 |
| **1958** | 10.49468 | 0.00027 | 7.130E-09 | 0.07574 | 10.49468 | 0.00027 | 7.130E-09 | 0.07574 |
| **1959** | 10.27100 | 0.00028 | 7.004E-09 | 0.08297 | 10.27100 | 0.00028 | 7.004E-09 | 0.08429 |
| **1960** | 10.27886 | 0.00027 | 7.060E-09 | 0.07714 | 10.27886 | 0.00027 | 7.060E-09 | 0.07612 |
| **1961** | 10.72697 | 0.00027 | 7.145E-09 | 0.06917 | 10.72697 | 0.00027 | 7.145E-09 | 0.06998 |
| **1962** | 10.98704 | 0.00027 | 7.079E-09 | 0.06945 | 10.98704 | 0.00027 | 7.079E-09 | 0.06841 |
| **1963** | 10.48942 | 0.00027 | 7.083E-09 | 0.08438 | 10.48942 | 0.00027 | 7.083E-09 | 0.08556 |
| **1964** | 9.43103 | 0.00028 | 6.985E-09 | 0.08217 | 9.43103 | 0.00028 | 6.985E-09 | 0.08052 |
| **1965** | 9.40720 | 0.00028 | 7.043E-09 | 0.08765 | 9.40720 | 0.00028 | 7.043E-09 | 0.08844 |
| **1966** | 10.26019 | 0.00027 | 7.157E-09 | 0.07052 | 10.26019 | 0.00027 | 7.157E-09 | 0.07073 |
| **1967** | 9.20724 | 0.00028 | 7.129E-09 | 0.08139 | 9.20724 | 0.00028 | 7.129E-09 | 0.08071 |
| **1968** | 9.34176 | 0.00028 | 7.249E-09 | 0.07917 | 9.34176 | 0.00028 | 7.249E-09 | 0.07686 |
| **1969** | 10.09088 | 0.00028 | 7.309E-09 | 0.07815 | 10.09088 | 0.00028 | 7.309E-09 | 0.07869 |
| **1970** | 11.27909 | 0.00027 | 7.384E-09 | 0.06820 | 11.27909 | 0.00027 | 7.384E-09 | 0.06766 |
| **1971** | 11.07327 | 0.00027 | 7.292E-09 | 0.07733 | 11.07327 | 0.00027 | 7.292E-09 | 0.07733 |
| **1972** | 10.26481 | 0.00027 | 7.241E-09 | 0.07424 | 10.26481 | 0.00027 | 7.241E-09 | 0.07286 |
| **1973** | 10.96316 | 0.00027 | 7.406E-09 | 0.06320 | 10.96316 | 0.00027 | 7.406E-09 | 0.06320 |
| **1974** | 10.80453 | 0.00027 | 7.358E-09 | 0.06994 | 10.80453 | 0.00027 | 7.358E-09 | 0.06994 |
| **1975** | 10.44231 | 0.00027 | 7.333E-09 | 0.08462 | 10.44231 | 0.00027 | 7.333E-09 | 0.08462 |
| **1976** | 9.82146 | 0.00028 | 7.297E-09 | 0.08321 | 9.82146 | 0.00028 | 7.297E-09 | 0.08336 |
| **1977** | 10.80680 | 0.00027 | 7.567E-09 | 0.07993 | 10.80680 | 0.00027 | 7.567E-09 | 0.07993 |
| **1978** | 11.24630 | 0.00027 | 7.541E-09 | 0.07030 | 11.24630 | 0.00027 | 7.541E-09 | 0.07030 |
| **1979** | 11.21259 | 0.00027 | 7.566E-09 | 0.08631 | 11.21259 | 0.00027 | 7.566E-09 | 0.08631 |
| **1980** | 10.75573 | 0.00027 | 7.549E-09 | 0.07822 | 10.75573 | 0.00027 | 7.549E-09 | 0.07822 |
| **1981** | 9.99192 | 0.00028 | 7.439E-09 | 0.08925 | 9.99192 | 0.00028 | 7.439E-09 | 0.08925 |
| **1982** | 10.90155 | 0.00027 | 7.643E-09 | 0.08452 | 10.90155 | 0.00027 | 7.643E-09 | 0.08452 |
| **1983** | 11.45528 | 0.00027 | 7.662E-09 | 0.07092 | 11.45528 | 0.00027 | 7.662E-09 | 0.07092 |
| **1984** | 11.50067 | 0.00027 | 7.623E-09 | 0.07006 | 11.50067 | 0.00027 | 7.623E-09 | 0.07006 |
| **1985** | 10.72531 | 0.00027 | 7.533E-09 | 0.08428 | 10.72531 | 0.00027 | 7.533E-09 | 0.08428 |
| **1986** | 10.63089 | 0.00027 | 7.600E-09 | 0.08232 | 10.63089 | 0.00027 | 7.600E-09 | 0.08232 |
| **1987** | 11.00111 | 0.00027 | 7.811E-09 | 0.07669 | 11.00111 | 0.00027 | 7.811E-09 | 0.07669 |
| **1988** | 11.17881 | 0.00027 | 7.728E-09 | 0.07656 | 11.17881 | 0.00027 | 7.728E-09 | 0.07608 |
| **1989** | 10.02154 | 0.00028 | 7.677E-09 | 0.09247 | 10.02154 | 0.00028 | 7.677E-09 | 0.09247 |
| **1990** | 9.78274 | 0.00028 | 7.699E-09 | 0.09597 | 9.78274 | 0.00028 | 7.699E-09 | 0.09597 |
| **1991** | 10.38632 | 0.00027 | 7.865E-09 | 0.08673 | 10.38632 | 0.00027 | 7.865E-09 | 0.08784 |
| **1992** | 10.57249 | 0.00027 | 7.883E-09 | 0.07859 | 10.57249 | 0.00027 | 7.883E-09 | 0.07859 |
| **1993** | 10.26603 | 0.00027 | 7.855E-09 | 0.08625 | 10.26603 | 0.00027 | 7.855E-09 | 0.08625 |
| **1994** | 9.85351 | 0.00028 | 7.826E-09 | 0.07911 | 9.85351 | 0.00028 | 7.826E-09 | 0.08243 |
| **1995** | 9.74136 | 0.00028 | 7.826E-09 | 0.07231 | 9.74136 | 0.00028 | 7.826E-09 | 0.09440 |
| **1996** | 11.11631 | 0.00027 | 8.050E-09 | 0.06808 | 11.11631 | 0.00027 | 8.050E-09 | 0.06898 |
| **1997** | 11.69731 | 0.00027 | 8.129E-09 | 0.07118 | 11.69731 | 0.00027 | 8.129E-09 | 0.06316 |
| **1998** | 11.34393 | 0.00027 | 8.047E-09 | 0.07775 | 11.34393 | 0.00027 | 8.047E-09 | 0.07524 |
| **1999** | 10.23738 | 0.00027 | 7.952E-09 | 0.09150 | 10.23738 | 0.00027 | 7.952E-09 | 0.08902 |
| **2000** | 10.25857 | 0.00027 | 7.980E-09 | 0.08666 | 10.25857 | 0.00027 | 7.980E-09 | 0.08025 |
| **2001** | 11.08866 | 0.00027 | 8.204E-09 | 0.06935 | 11.08866 | 0.00027 | 8.204E-09 | 0.06702 |
| **2002** | 11.51782 | 0.00027 | 8.168E-09 | 0.07470 | 11.51782 | 0.00027 | 8.168E-09 | 0.06493 |
| **2003** | 11.19079 | 0.00027 | 8.201E-09 | 0.08222 | 11.19079 | 0.00027 | 8.201E-09 | 0.07161 |
| **2004** | 10.07263 | 0.00027 | 8.110E-09 | 0.07709 | 10.07263 | 0.00027 | 8.110E-09 | 0.08182 |
| **2005** | 10.82111 | 0.00027 | 8.308E-09 | 0.07016 | 10.82111 | 0.00027 | 8.308E-09 | 0.07845 |
| **2006** | 10.67874 | 0.00027 | 8.360E-09 | 0.09330 | 10.39232 | 0.00027 | 8.286E-09 | 0.08103 |
| **2007** | 11.20212 | 0.00027 | 8.543E-09 | 0.07093 | 10.97929 | 0.00027 | 8.500E-09 | 0.07285 |
| **2008** | 10.97773 | 0.00027 | 8.512E-09 | 0.07356 | 10.38860 | 0.00027 | 8.402E-09 | 0.07189 |
| **2009** | 10.32496 | 0.00027 | 8.379E-09 | 0.07534 | 10.05480 | 0.00028 | 8.395E-09 | 0.08427 |
| **2010** | 11.19133 | 0.00027 | 8.546E-09 | 0.08113 | 10.44428 | 0.00027 | 8.517E-09 | 0.07245 |
| **2011** | 10.74789 | 0.00027 | 8.573E-09 | 0.08212 | 11.00538 | 0.00027 | 8.660E-09 | 0.06779 |
| **2012** | 10.78375 | 0.00027 | 8.613E-09 | 0.07427 | 10.45286 | 0.00027 | 8.546E-09 | 0.07624 |
| **2013** | 10.79581 | 0.00027 | 8.604E-09 | 0.08114 | 10.43815 | 0.00027 | 8.606E-09 | 0.08400 |
| **2014** | 10.89822 | 0.00027 | 8.701E-09 | 0.08271 | 10.77177 | 0.00027 | 8.685E-09 | 0.07985 |
| **2015** | 11.74382 | 0.00027 | 8.785E-09 | 0.07984 | 12.05516 | 0.00026 | 8.898E-09 | 0.06260 |
| **2016** | 11.22115 | 0.00027 | 8.757E-09 | 0.07737 | 10.73437 | 0.00027 | 8.686E-09 | 0.07725 |
| **2017** | 10.64904 | 0.00027 | 8.822E-09 | 0.08190 | 10.64482 | 0.00027 | 8.907E-09 | 0.07938 |
| **2018** | 12.03004 | 0.00026 | 9.080E-09 | 0.09797 | 10.49908 | 0.00027 | 8.908E-09 | 0.07603 |
| **2019** | 11.63216 | 0.00027 | 8.937E-09 | 0.08273 | 10.10589 | 0.00027 | 8.883E-09 | 0.08736 |
| **2020** | 10.99267 | 0.00027 | 8.990E-09 | 0.07904 | 10.33919 | 0.00028 | 9.047E-09 | 0.07885 |
| **2021** | 10.77191 | 0.00027 | 8.983E-09 | 0.07910 | 11.72108 | 0.00027 | 9.224E-09 | 0.06373 |
| **2022** | 10.08023 | 0.00028 | 8.955E-09 | 0.09592 | 11.74443 | 0.00027 | 9.127E-09 | 0.06160 |
| **2023** | 10.93052 | 0.00027 | 9.230E-09 | 0.08698 | 11.74647 | 0.00027 | 9.123E-09 | 0.07645 |
| **2024** | 10.83859 | 0.00027 | 9.198E-09 | 0.08093 | 11.41938 | 0.00027 | 9.129E-09 | 0.07399 |
| **2025** | 11.76092 | 0.00027 | 9.324E-09 | 0.08005 | 11.81962 | 0.00027 | 9.312E-09 | 0.08296 |
| **2026** | 12.39285 | 0.00026 | 9.302E-09 | 0.07136 | 11.75634 | 0.00027 | 9.331E-09 | 0.07765 |
| **2027** | 10.91983 | 0.00027 | 9.107E-09 | 0.09461 | 10.73476 | 0.00027 | 9.302E-09 | 0.07815 |
| **2028** | 11.44136 | 0.00027 | 9.483E-09 | 0.09291 | 11.45032 | 0.00027 | 9.597E-09 | 0.06822 |
| **2029** | 10.80388 | 0.00027 | 9.316E-09 | 0.08433 | 12.06396 | 0.00027 | 9.699E-09 | 0.07975 |
| **2030** | 11.35546 | 0.00027 | 9.521E-09 | 0.08031 | 11.19723 | 0.00027 | 9.656E-09 | 0.07956 |
| **2031** | 11.25269 | 0.00027 | 9.402E-09 | 0.07769 | 11.04332 | 0.00027 | 9.704E-09 | 0.08978 |
| **2032** | 10.99961 | 0.00027 | 9.413E-09 | 0.09126 | 11.19877 | 0.00027 | 9.870E-09 | 0.08333 |
| **2033** | 10.46201 | 0.00027 | 9.428E-09 | 0.08358 | 9.91200 | 0.00028 | 9.688E-09 | 0.10052 |
| **2034** | 11.33826 | 0.00027 | 9.511E-09 | 0.07704 | 11.00090 | 0.00027 | 1.006E-08 | 0.07729 |
| **2035** | 11.63019 | 0.00027 | 9.600E-09 | 0.07399 | 11.14413 | 0.00027 | 1.011E-08 | 0.07959 |
| **2036** | 11.04913 | 0.00027 | 9.530E-09 | 0.08367 | 10.63204 | 0.00027 | 9.954E-09 | 0.10281 |
| **2037** | 10.55131 | 0.00027 | 9.500E-09 | 0.09148 | 11.01380 | 0.00027 | 1.018E-08 | 0.08501 |
| **2038** | 10.24276 | 0.00027 | 9.397E-09 | 0.09157 | 10.86756 | 0.00027 | 1.030E-08 | 0.07708 |
| **2039** | 11.48969 | 0.00027 | 9.632E-09 | 0.08534 | 11.31408 | 0.00027 | 1.038E-08 | 0.06417 |
| **2040** | 11.44680 | 0.00027 | 9.501E-09 | 0.08269 | 10.65756 | 0.00027 | 1.035E-08 | 0.08531 |
| **2041** | 11.77623 | 0.00027 | 9.739E-09 | 0.07330 | 10.37384 | 0.00028 | 1.053E-08 | 0.08152 |
| **2042** | 11.68783 | 0.00027 | 9.645E-09 | 0.08929 | 11.34380 | 0.00027 | 1.076E-08 | 0.07204 |
| **2043** | 10.89068 | 0.00027 | 9.557E-09 | 0.08929 | 11.72140 | 0.00027 | 1.084E-08 | 0.07671 |
| **2044** | 10.90700 | 0.00027 | 9.597E-09 | 0.09068 | 12.36015 | 0.00026 | 1.095E-08 | 0.07413 |
| **2045** | 11.01395 | 0.00027 | 9.780E-09 | 0.06653 | 12.45932 | 0.00026 | 1.093E-08 | 0.07111 |
| **2046** | 11.34731 | 0.00027 | 9.754E-09 | 0.07422 | 12.49620 | 0.00026 | 1.102E-08 | 0.06491 |
| **2047** | 11.96595 | 0.00026 | 9.802E-09 | 0.07239 | 11.27586 | 0.00027 | 1.091E-08 | 0.10055 |
| **2048** | 11.42797 | 0.00027 | 9.609E-09 | 0.07307 | 12.47177 | 0.00026 | 1.135E-08 | 0.07431 |
| **2049** | 11.00117 | 0.00027 | 9.577E-09 | 0.07254 | 12.28186 | 0.00026 | 1.134E-08 | 0.06656 |
| **2050** | 10.30267 | 0.00027 | 9.543E-09 | 0.27143 | 11.60490 | 0.00027 | 1.140E-08 | 0.08320 |
| **2051** | 10.69664 | 0.00027 | 9.775E-09 | 0.08755 | 12.50424 | 0.00026 | 1.162E-08 | 0.07060 |
| **2052** | 11.26148 | 0.00027 | 9.715E-09 | 0.12853 | 12.09657 | 0.00027 | 1.156E-08 | 0.09060 |
| **2053** | 11.71809 | 0.00027 | 9.821E-09 | 0.07339 | 10.88858 | 0.00027 | 1.176E-08 | 0.08242 |
| **2054** | 12.39312 | 0.00026 | 9.737E-09 | 0.17206 | 11.80507 | 0.00027 | 1.207E-08 | 0.06964 |
| **2055** | 11.74378 | 0.00027 | 9.645E-09 | 0.17347 | 11.21590 | 0.00027 | 1.215E-08 | 0.08030 |
| **2056** | 12.28731 | 0.00026 | 9.771E-09 | 0.07877 | 11.21637 | 0.00027 | 1.214E-08 | 0.08397 |
| **2057** | 11.77286 | 0.00027 | 9.663E-09 | 0.07922 | 12.22559 | 0.00026 | 1.237E-08 | 0.07245 |
| **2058** | 11.21999 | 0.00027 | 9.644E-09 | 0.07876 | 12.03513 | 0.00026 | 1.240E-08 | 0.07922 |
| **2059** | 11.61379 | 0.00027 | 9.695E-09 | 0.07949 | 11.89397 | 0.00027 | 1.263E-08 | 0.08133 |
| **2060** | 11.39991 | 0.00027 | 9.777E-09 | 0.08952 | 12.04298 | 0.00027 | 1.273E-08 | 0.09305 |
| **2061** | 11.43837 | 0.00027 | 9.692E-09 | 0.08823 | 11.01351 | 0.00027 | 1.273E-08 | 0.09316 |
| **2062** | 11.88947 | 0.00027 | 9.796E-09 | 0.08502 | 11.91862 | 0.00027 | 1.302E-08 | 0.08327 |
| **2063** | 10.30219 | 0.00028 | 9.589E-09 | 0.08503 | 12.12393 | 0.00026 | 1.318E-08 | 0.07306 |
| **2064** | 10.89860 | 0.00027 | 9.656E-09 | 0.10488 | 12.06545 | 0.00026 | 1.333E-08 | 0.07903 |
| **2065** | 11.39859 | 0.00027 | 9.666E-09 | 0.09142 | 10.76791 | 0.00027 | 1.335E-08 | 0.09178 |
| **2066** | 10.61478 | 0.00027 | 9.526E-09 | 0.08902 | 10.62458 | 0.00027 | 1.358E-08 | 0.08391 |
| **2067** | 10.97466 | 0.00027 | 9.655E-09 | 0.08475 | 12.24319 | 0.00027 | 1.390E-08 | 0.07797 |
| **2068** | 10.53774 | 0.00027 | 9.621E-09 | 0.08219 | 11.14941 | 0.00027 | 1.380E-08 | 0.08700 |
| **2069** | 11.07424 | 0.00027 | 9.736E-09 | 0.09073 | 12.72501 | 0.00026 | 1.416E-08 | 0.07663 |
| **2070** | 11.57530 | 0.00027 | 9.740E-09 | 0.08318 | 12.75731 | 0.00026 | 1.421E-08 | 0.07298 |
| **2071** | 10.37480 | 0.00027 | 9.618E-09 | 0.08332 | 11.66557 | 0.00027 | 1.422E-08 | 0.08472 |
| **2072** | 10.44155 | 0.00028 | 9.579E-09 | 0.09614 | 11.47543 | 0.00027 | 1.448E-08 | 0.08861 |
| **2073** | 10.76128 | 0.00027 | 9.680E-09 | 0.08428 | 12.00138 | 0.00027 | 1.480E-08 | 0.08052 |
| **2074** | 12.32818 | 0.00026 | 9.768E-09 | 0.08381 | 11.56169 | 0.00027 | 1.497E-08 | 0.08101 |
| **2075** | 12.17414 | 0.00026 | 9.596E-09 | 0.08555 | 11.76610 | 0.00027 | 1.510E-08 | 0.08963 |
| **2076** | 11.46326 | 0.00027 | 9.520E-09 | 0.08459 | 11.92884 | 0.00027 | 1.523E-08 | 0.08530 |
| **2077** | 12.05341 | 0.00026 | 9.565E-09 | 0.08164 | 12.85895 | 0.00026 | 1.544E-08 | 0.07896 |
| **2078** | 11.95087 | 0.00026 | 9.573E-09 | 0.08105 | 13.27250 | 0.00026 | 1.568E-08 | 0.07164 |
| **2079** | 11.64245 | 0.00027 | 9.544E-09 | 0.08774 | 12.12377 | 0.00026 | 1.565E-08 | 0.08492 |
| **2080** | 10.82251 | 0.00027 | 9.408E-09 | 0.08788 | 12.05957 | 0.00027 | 1.583E-08 | 0.09394 |
| **2081** | 10.86875 | 0.00027 | 9.489E-09 | 0.08706 | 13.05582 | 0.00026 | 1.615E-08 | 0.07112 |
| **2082** | 11.92243 | 0.00027 | 9.636E-09 | 0.07671 | 12.60556 | 0.00026 | 1.615E-08 | 0.08233 |
| **2083** | 12.41736 | 0.00026 | 9.569E-09 | 0.07016 | 12.11144 | 0.00027 | 1.645E-08 | 0.08249 |
| **2084** | 10.90232 | 0.00027 | 9.365E-09 | 0.06974 | 12.19577 | 0.00027 | 1.660E-08 | 0.08664 |
| **2085** | 11.54092 | 0.00027 | 9.510E-09 | 0.07352 | 12.73392 | 0.00026 | 1.689E-08 | 0.07345 |
| **2086** | 11.00988 | 0.00027 | 9.330E-09 | 0.08329 | 13.43082 | 0.00026 | 1.707E-08 | 0.07619 |
| **2087** | 11.47979 | 0.00027 | 9.531E-09 | 0.08717 | 12.86245 | 0.00026 | 1.692E-08 | 0.09307 |
| **2088** | 10.82586 | 0.00027 | 9.401E-09 | 0.08638 | 11.88503 | 0.00027 | 1.719E-08 | 0.09932 |
| **2089** | 12.19913 | 0.00026 | 9.648E-09 | 0.08053 | 12.21087 | 0.00027 | 1.747E-08 | 0.09742 |
| **2090** | 12.00074 | 0.00026 | 9.482E-09 | 0.07529 | 12.29405 | 0.00026 | 1.755E-08 | 0.08919 |
| **2091** | 11.30762 | 0.00027 | 9.347E-09 | 0.07618 | 12.43185 | 0.00026 | 1.788E-08 | 0.08548 |
| **2092** | 11.01687 | 0.00027 | 9.316E-09 | 0.07626 | 11.47820 | 0.00027 | 1.792E-08 | 0.09410 |
| **2093** | 11.12328 | 0.00027 | 9.399E-09 | 0.08311 | 12.07953 | 0.00027 | 1.838E-08 | 0.08161 |
| **2094** | 11.23065 | 0.00027 | 9.451E-09 | 0.08272 | 12.54175 | 0.00026 | 1.851E-08 | 0.07993 |
| **2095** | 10.26100 | 0.00028 | 9.281E-09 | 0.08966 | 12.77308 | 0.00026 | 1.854E-08 | 0.08270 |
| **2096** | 10.77382 | 0.00027 | 9.397E-09 | 0.08966 | 13.00663 | 0.00026 | 1.877E-08 | 0.08431 |
| **2097** | 11.82491 | 0.00027 | 9.442E-09 | 0.06892 | 13.96841 | 0.00026 | 1.905E-08 | 0.07230 |
| **2098** | 11.39923 | 0.00027 | 9.286E-09 | 0.07103 | 13.89398 | 0.00026 | 1.913E-08 | 0.07448 |
| **2099** | 10.86269 | 0.00027 | 9.248E-09 | 0.08929 | 12.81535 | 0.00026 | 1.926E-08 | 0.08432 |

**References**

1. Field, J. C. Application of ecosystem-based fishery management approaches in the northern California Current. PhD thesis, University of Washington, Seattle, WA, USA. 408 pp. (2004).
2. Lachmuth, C.L., Alava, J.J., Hickie, B.E., Johannessen, S.C., Macdonald, R.W., Ford, J.K.B., *et al*. Ocean disposal in resident killer whale (*Orcinus orca*) critical habitat: science in support of risk management. Fisheries and Oceans Canada, DFO*. Can. Sci. Advis. Sec. Res. Doc*. **116**, 1–172 (2010).
3. Alava, J.J., Ross, P.S., Lachmuth, C.L., Ford, J.K.B., Hickie, B. & Gobas, F.A.P.C. Habitat based PCB environmental quality criteria for the protection of endangered killer whales (*Orcinus orca*). *Environ. Sci. Technol.* **46**, 12655–12663 (2012).
4. Alava, J.J., Ross, P.S. & Gobas, A.P.C. Food web bioaccumulation model for resident killer whales from the Northeastern Pacific Ocean as a tool for the derivation of PBDE-Sediment Quality Guidelines. *Arch. Environ. Contam. Toxicol.* **70**(1), 155-168 (2016).
5. Ford, J.K.B. & Ellis, G.M. Selective foraging by fish-eating killer whales Orcinus orca in British Columbia. *Mar. Ecol. Prog. Ser.* **316**, 185–199 (2006).
6. Ford, J.K.B., Wright, B.M., Ellis, G.M. & Candy, J.R. Chinook salmon predation by resident killer whales: seasonal and regional selectivity, stock identity of prey, and consumption rates. Pacific Biological Station, Fisheries and Oceans Canada, DFO. *Can. Sci. Advis. Sec. Res. Doc.* **101**, 1–43 (2009).
7. Ford, J.K.B., Ellis, G.M., Olesiuk, P.F. & Balcomb, K.C. Linking killer whale survival and prey abundance: food limitation in the oceans' apex predator? *Biol. Lett.* **6**, 139–142 (2010). <http://dx.doi.org/10.1098/rsbl.2009.0468>.
8. Alava, J.J. & Gobas, F.A. Modeling 137 Cs bioaccumulation in the salmon–resident killer whale food web of the Northeastern Pacific following the Fukushima Nuclear Accident. *Sci. Total Environ.* **544**, 56-67 (2016).
9. Dunne, J.P. *et al*. GFDL's ESM2 global coupled climate–carbon earth systemmodels. Part I: physical formulation and baseline simulation characteristics. *J. Clim.* **25**, 6646–6665 (2012).
10. Guénette, S., Araújo, J.N. & Bundy, A. Exploring the potential effects of climate change on the Western Scotian Shelf ecosystem, Canada. *J. Mar. Syst.* **134**, 89–100 (2014).
11. Ainsworth, C.H., Samhouri, J.F., Busch, D.S., Cheung, W.W.L., Dunne, J. & Okey, T.A. Potential impacts of climate change on Northeast Pacific marine foodwebs and fisheries. *ICES* *J. Mar. Sci.* **68**, 1217–1229 (2011)
12. Cummins, P. & Masson, D. Trends in sea surface temperature and salinity. in *Climate Trends and Projections for the Pacific Large Aquatic Basin* (eds. Christian, J. R. & Foreman, M.G.G.)*.* *Can. Tech. Rep. Fish. Aquat. Sci*. 3032, 4-10 (2013).
13. IPCC. Climate Change 2014: Impacts, Adaptation, and Vulnerability, *in* *Part B: Regional Aspects. Contribution of Working Group II to the Fifth Assessment Report of the Intergovernmental Panel on Climate Change* (eds. Barros, V.R. *et al*.). pp. 688 (Cambridge University Press, Cambridge,United Kingdom and New York, NY, USA, 2014).
14. Okey, T.A., Alidina, H.M., Lo, V. & Jessen, S. Effects of climate change on Canada's Pacific marine ecosystems: a summary of scientific knowledge. *Rev. Fish. Biol. Fish.* 24, 519e559 (2014). <http://dx.doi.org/10.1007/s11160-014-9342-1>.
15. BCME. Environmental trends in British Columbia: 2007. State of environment reporting. (B.C. Ministry of Environment, Victoria, B.C., 2007) [www.env.gov.bc.ca/soe/et07/](http://www.env.gov.bc.ca/soe/et07/)
16. Beamish, R.J., King, J.R. & McFarlane G.A. Canada, in *Impacts of climate and climate change on the key species in the fisheries in the North Pacific*. PICES working group on climate change, shifts in fish populations, and fisheries management. North Pacific Marine Science Organization (PICES). PICES scientific report no. 35. (ed. Beamish, R.J.) 14–55, (Secretariat, Sidney, BC, 2009).
17. Chandler, P. Sea surface temperature and salinity trends observed at lighthouses and weather buoys in British Columbia, 2015, in *State of the physical, biological and selected fishery resources of Pacific Canadian marine ecosystems in 2015* (eds. Chandler, P.C., King, S.A., and Perry, R.I). *Can. Tech. Rep. Fish. Aquat. Sci.* 3179, viii + 230 p. (2016)
18. Sarmiento, J. L., Hughes, T. M. C., Stouffer, R. J. & Manabe, S. Simulated response of the ocean carbon cycle to anthropogenic climate warming. *Nature* 393: 245–249 (1998).
19. Keeling, R. F., Kortzinger, A. & Gruber, N. Ocean deoxygenation in a warming world. *Ann. Rev. Mar. Sci.,* **2**, 199–229 (2010).
20. Rabalais, N.N., Diaz, R.J., Levin, L.A., Turner, R.E., Gilbert, D. & Zhang, J. Dynamics and distribution of natural and human caused hypoxia. *Biogeosciences* 7(2), 585–619 (2010).
21. Batten, S. *et al*. Status and trends of the North Pacific oceanic region, 2003–2008, in *Marine ecosystems of the North Pacific Ocean*, *2003–2008* (eds. McKinnell, S.M. & Dagg M.J.). *PICES* *Spec. Publ.* 4, 56–105 (2010).
22. Whitney, F.A., Freeland, H.J. & Robert, M. Persistently declining oxygen levels in the interior waters of the eastern subarctic Pacific. *Prog. Oceanogr*. **75**(2), 179–199 (2007).
23. Crawford, W.R. & Peña, M.A. Declining oxygen on the British Columbia Continental Shelf. *Atmos. Ocean.* **51**(1), 88–103 (2013).
24. Crawford, W.R. & Peña, M.A. Trends in oxygen concentration on the continental shelf of British Columbia, in *Climate Trends and Projections for the Pacific Large Aquatic Basin* (eds. Christian, J. R. & Foreman, M.G.G*.*)*.* 46-53. *Can. Tech. Rep. Fish. Aquat. Sci*. 3032, xi + 112 p. (2013).
25. Feely, R.A., Sabine, C.L., Hernandez-Ayon, J.M., Ianson, D. & Hales, B. Evidence for upwelling of corrosive ‘‘acidified’’ water onto the continental shelf. *Science* 320:1490–1492 (2008).
26. Hauri, C., Gruber, N., Plattner, G-K., Alin, S., Feely, R.A., Hales, B. & Wheeler, P.A. Ocean acidification in the California current system. Oceanography **22**(4), 61–71 (2009).
27. Peña, A. & N. Nemcek. Phytoplankton in the Surface Waters along Line P and Off the West Coast of Vancouver Island, in *State of the physical, biological and selected fishery resources of Pacific Canadian marine ecosystems in 2015* (eds. Chandler, P.C., King, S.A. & Perry, R.I.). *Can. Tech. Rep. Fish. Aquat. Sci*. 3179, viii + 230 p (2016).
28. Hare, C.E., Leblanc, K., DiTullio, G.R., Kudela, R.M., Zhang, Y., Lee, P.A., Riseman, S. & Hutchins D.A. Consequences of increased temperature and CO2 for phytoplankton community structure in the Bering Sea. *Mar. Ecol. Prog. Ser.* **352**, 9–16 (2007).
29. Richardson, A.J. In hot water: zooplankton and climate change. *ICES J. Mar. Sci.* **65**(3), 279–295 (2008).
30. Johannessen, S.C., Macdonald, R.W., Wright, C.A., Burd, B., Shaw, D.P. & Van Roodselaar A. Joined by geochemistry, divided by history: PCBs and PBDEs in Strait of Georgia sediments. *Mar. Environ. Res.* **66**, S112–S120 (2008).
31. Johannessen, S.C., Macdonald, R.W. and Eek, M.K.. Historical trends in mercury sedimentation and mixing in the Strait of Georgia, Canada. *Environ. Sci. Technol.* **39**, 4361–4368 (2005).
32. Macdonald, R.W., Cretney, W.J., Crewe, N. & Paton, D. A history ofoctachlorodibenzo-*p*-dioxin, 2,3,7,8-tetrachlorodibenzofuran, and 3,3',4,4'-tetrachlorobiphenyl contamination in Howe Sound, British Columbia. *Environ. Sci. Technol.* **26**, 1544-1550 (1992).
33. Ross, P.S., Ellis, G.M., Ikonomou, M.G., Barrett-Lennard, L.G., Addison RF. High PCB concentrations in free-ranging Pacific killer whales, *Orcinus orca*: Effects of age, sex and dietary preference. *Mar. Pollut. Bull.* **40**, 504-515 (2000).
34. Cullon, D.L., Jeffries, S.J. & Ross, P.S. Persistent organic pollutants in the diet of harbor seals (*Phoca vitulina*) inhabiting Puget Sound, Washington (USA), and the Strait of Georgia, British Columbia (Canada): a food basket approach. *Environ. Toxicol. Chem.* **24**(10), 2562-2572 (2005).
35. Krahn, M.M., Hanson, M.B., Baird, R.W., Boyer, R.H., Burrows, D.G., Emmons, C.K., Ford, J.K., Jones, L.L., Noren, D.P., Ross, P.S. & Schorr, G.S. Persistent organic pollutants and stable isotopes in biopsy samples (2004/2006) from Southern Resident killer whales. *Mar. Pollut. Bull.*  **54**(12), 1903-1911 (2007).
36. Cullon, D. L., Yunker, M. B., Alleyne, C., Dangerfield, N. J., O’Neill, S., Whiticar, M. J., & Ross, P. S. Persistent organic pollutants in Chinook salmon (*Oncorhynchus tshawytscha*): Implications for resident killer whales of British Columbia and adjacent waters. *Environ. Toxicol. Chem.* **28**, 148–161 (2009).
37. Buckman, A.H., Veldhoen, N., Ellis, G., Ford, J.K.B., Helbing, C., & Ross, P.S. PCB-associated changes in mRNA expression in killer whales (*Orcinus or*ca ) from the NE Pacific Ocean. *Environ. Sci. Technol.* **45**, 10194–10202 (2011).
38. Alava, J.J., Lambourn, D., Olesiuk, P., Lance, M., Jeffries, S.J., Gobas, F.A. & Ross, P.S. PBDE flame retardants and PCBs in migrating Steller sea lions (*Eumetopias jubatus*) in the Strait of Georgia, British Columbia, Canada. *Chemosphere*, **88**(7), 855-864 (2012).
39. Frouin, H., Dangerfield, N., Macdonald, R.W., Galbraith, M., Crewe, N., Shaw, P., Mackas, D. & Ross PS. Partitioning and bioaccumulation of PCBs and PBDEs in marine plankton from Strait of Georgia, British Columbia, Canada. *Prog. Oceanogr*. **115**, 65–75 (2013).
40. Ross, P.S., Noël, M., Lambourn, D., Dangerfield, N., Calambokidis, J. &J effries, S. Declining concentrations of persistent PCBs, PBDEs, PCDEs, and PCNs in harbor seals (*Phoca vitulina*) from the Salish Sea. *Prog. Oceanogr*. **115**, 160-170 (2013).
41. Grant, P.B.C., Johannessen, S.C., Macdonald, R.W., Yunker, M., Sanborn, M., Dangerfield, N., Wright, C., Ross, P.S. Environmental fractionation of PCBs and PBDEs during particle transport as recorded by sediments in coastal waters. *Environ. Toxicol. Chem.* 30, 1522–1532 (2011).
42. Kelly, B.C., Ikonomou, M.G., Higgs, D.A., Oakes, J., Dubetz, C. Mercury and other trace elements in farmed and wild salmon from British Columbia, Canada. *Environ. Toxicol. Chem.* **27**(6), 1361-70 (2008).
43. Noël, M., Jeffries, S., Lambourn, D.M., Telmer, K., Macdonald, R. & Ross, P.S. Mercury accumulation in harbour seals from the northeastern Pacific Ocean: the role of transplacental transfer, lactation, age and location. *Arch. Environ. Contam. Toxicol*. **70**(1), 56-66 (2016)
44. Booth, S. & Zeller, D. Mercury, Food Webs, and Marine Mammals: Implications of Diet and Climate Change for Human Health. *Environ. Health Perspect.* **113**(5), 521-526 (2005).
45. Christensen, V. & Walters, C.J. Ecopath with Ecosim: methods, capabilities and limitations. *Ecol. Model.* **172**, 109–139 (2004).
46. Christensen, V. & Pauly, D. ECOPATH II—A software for balancing steady-state models and calculating network characteristics. *Ecol. Model.* **61**, 169–185 (1992).
47. Christensen, V., Walters, C. J. & Pauly, D. Ecopath with Ecosim: a User’s Guide. (Fisheries Centre, University of British Columbia, Canada. 154 pp, 2005).
48. Christensen, V., Walters, C., Pauly, D. & Forrest, R.. Ecopath with Ecosim Version6: User Guide. Lenfest Ocean Futures Project (2008).
49. Coombs, A.P. Marine mammals and human health in the eastern Bering Sea: Using an ecosystem-based food web model to track PCBs. (MSc Thesis, University of British Columbia, Vancouver, BC. 91p., 2004).
50. Booth, S. *et al*. Pollutants in the seas around us, in *Global Atlas of Marine Fisheries. A critical appraisal of catches and ecosystem impacts* (eds. Pauly, D. & Zeller, D.) 152-170. (Island Press, Washington, Covelo, London, 2016)
51. O’Neill, S. M., & West, J. E. Marine distribution, life history traits, and the accumulation of polychlorinated biphenyls in Chinook salmon from Puget Sound, Washington. *Trans Am Fish Soc.* **13**8, 616–632 (2009).
52. Lundin, J.I. *et al.* Modulation in persistent organic pollutant concentration and profile by prey availability and reproductive 916 status in Southern Resident killer whale scat samples. *Environ. Sci. Technol.* **50**, 6506-6516 (2016).
53. Hickie, B.E., Ross, P.S., Macdonald, R.W. & Ford, J.K.B. Killer whales (*Orcinus orca*) face protracted health risks associated with lifetime exposure to PCBs. *Environ. Sci. Technol.* **41**, 6613–6619 (2007).
54. Borga, K. *et al*. Trophic magnification factors: Considerations of ecology, ecosystems, and study design. *Integr. Environ. Assess. Manage*. **8**, 64−84 (2012).
55. Conder, J.M., Gobas, F.A., Borgå, K., Muir, D.C. and Powell, D.E. Use of trophic magnification factors and related measures to characterize bioaccumulation potential of chemicals. *Integr. Environ. Assess. Manage* **8**(1), 85-97 (2012).
56. Lavoie, R.A., Jardine, T.D., Chumchal, M.M., Kidd, K.A., Campbell, L.M. Biomagnification of mercury in aquatic food webs: a worldwide meta-analysis. *Environ. Sci. Technol.* **47**, 13385-13394 (2013).
57. Walters, D. M., Jardine, T. D., Cade, B. S., Kidd, K. A., Derek, C.G.M & Leipzig-Scott, P. Trophic magnification of organic chemicals: A global synthesis. *Environ. Sci. Technol.* **50**, 4650-4658 (2016).
58. World Health Organization (WHO). Environmental health criteria 101: Methylmercury, International Programme of Chemical Safety. (International Programme of Chemical Safety, Geneva 1990).
59. Alava, J. J., Cheung, W. W.L., Ross, P. S. & Sumaila, R. U. Climate change-contaminant interactions in marine food webs: Towards a conceptual framework. *Glob. Change Biol.* 23, 3984–4001 (2017).
60. Cisneros-Montemayor, A. M., Pauly, D., Weatherdon, L. V. & Ota, Y. A global estimate of seafood consumption by coastal Indigenous peoples. *PLoS ONE*, 11(12), e0166681. (2016) doi:10.1371/journal. pone.0166681.
61. Bradley, M. A., Barst, B. D. & Basu, N. A review of mercury bioavailability in humans and fish. *Int. J. Environ. Res. Public Health* *14*(2), 169. (2017).
